# Enhancing the solubility and potency of tetrahydrocurcumin as an anti-cancer agent using a β-cyclodextrin inclusion complex approach

**DOI:** 10.1371/journal.pone.0305171

**Published:** 2024-07-26

**Authors:** Zhi Xuan Low, Michelle Yee Mun Teo, Fariza Juliana Nordin, Vijayaraj Kumar Palanirajan, Beata Morak-Młodawska, Asma Saleem Qazi, Lionel Lian Aun In

**Affiliations:** 1 Department of Biotechnology, Faculty of Applied Sciences, UCSI University, Kuala Lumpur, Malaysia; 2 Department of Pharmaceutical Technology, Faculty of Pharmaceutical Sciences, UCSI University, Kuala Lumpur, Malaysia; 3 Department of Biological Sciences, National University of Medical Sciences, Rawalpindi, Pakistan; 4 Department of Biological Sciences and Biotechnology, Faculty of Science and Technology, Universiti Kebangsaan Malaysia, Bangi, Selangor, Malaysia; 5 Department of Organic Chemistry, Faculty of Pharmaceutical Sciences, Medical University of Silesia, Katowice, Poland; University of Brescia: Universita degli Studi di Brescia, ITALY

## Abstract

Curcuminoids originating from turmeric roots are renowned for their diverse pharmacological applications, particularly as a natural anticancer agent. Unfortunately, harnessing the full potential of curcumin derivatives in cancer therapy has been impeded by its inherent limitations, specifically instabilities owing to poor solubility, leading to low systemic bioavailability under normal physiological circumstances. To circumvent this, a novel organic-based drug delivery system employing physically adsorbed β-cyclodextrin (βCD) as an excipient was developed in this study. This resulted in improved aqueous dispersion coupled with anticancer enhancements of tetrahydrocurcumin (THC) at a molar ratio of 2:1. Encapsulation of this agent was confirmed by physicochemical characterisation using UV–vis spectroscopy, differential scanning calorimetry (DSC), Fourier transform infrared spectroscopy (FTIR), scanning electron microscopy (SEM), and both *in vitro* and *in vivo* approaches. Through the presence of an inclusion complex, a higher aqueous dispersion (65-fold) resulting in a higher drug content and an elevated release profile was achieved. Athymic nude (Nu/Nu) mice exposed to this treatment displayed improvements in tumour regression compared to stand-alone agents, consistent with *in vitro* cytotoxicity assays with an SI value > 10. The inclusion complex further enhanced apoptosis, as well as anti-migration and anti-invasion rates. Mechanistically, this formulation was consistent in terms of caspase 3 activation. Furthermore, the inclusion complex exhibited reduced systemic toxicity, including reduced inflammation in vital organs as examined by hematoxylin and eosin (H&E) staining. This study also revealed a notable sequential reduction in serum levels of tumour markers, including carcinoembryonic antigen (CEA) and mouse Cytochrome P450 1A2 (CYP1A2), correlating with a significant decrease in tumour bulk volume upon treatment commencement. These compelling findings highlight the potential of this formulation to empower insoluble or poorly soluble hydrophobic agents, thus offering promising prospects for their effective utilisation in colorectal cancer (CRC) chemotherapy.

## Introduction

**Fig 1:**
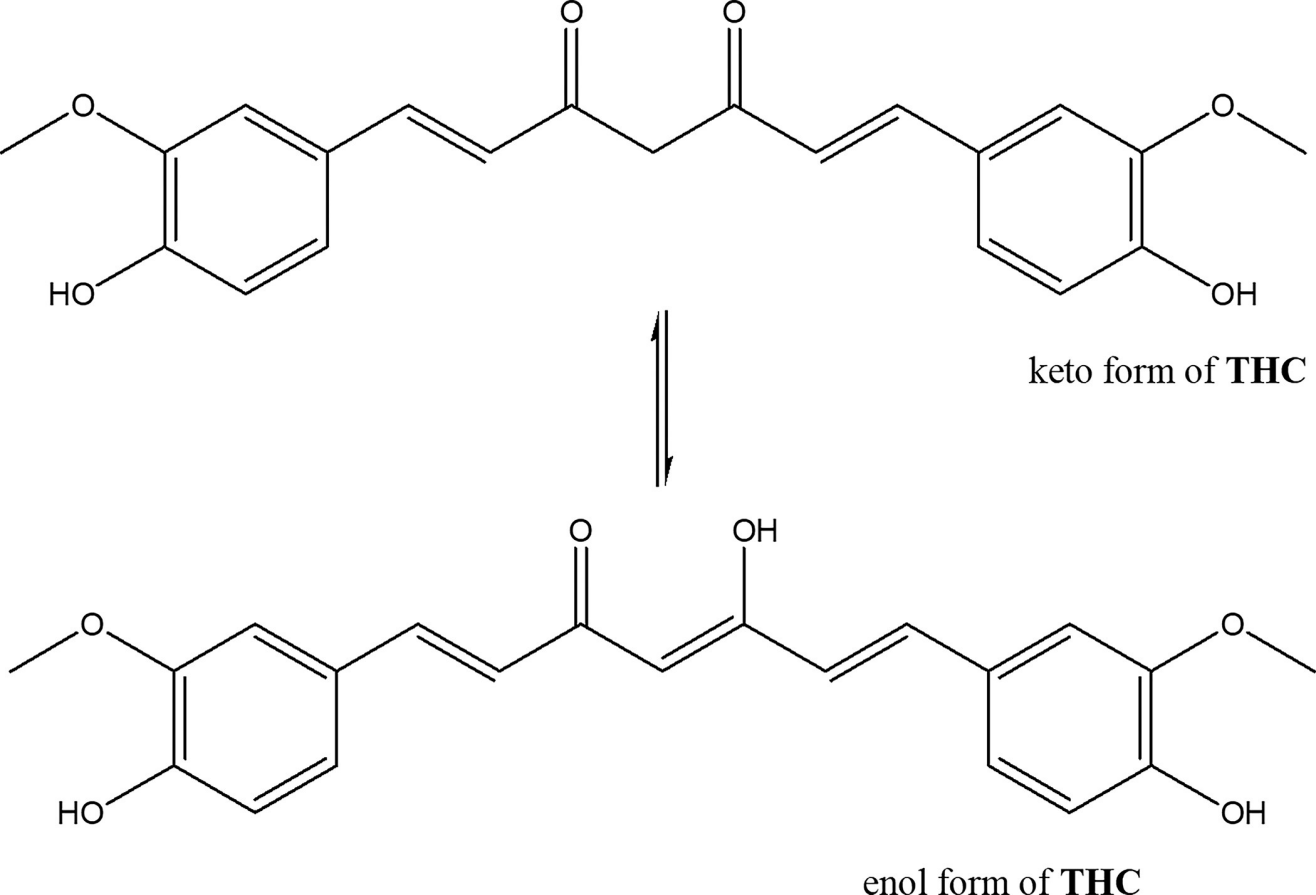
Structure of THC and its tautomeric structure.

Tetrahydrocurcumin (THC) is a major metabolite of a curcumin derivative obtained from the turmeric rhizome (*Curcuma longa Linn*.) plant belonging to the family *Zingiberaceae*. THC is produced after curcumin is reduced by an endogenous reductase system, underlying similar structural features with distinct chemical characteristics and biological properties [1–3]. The THC structure is presented in Fig 1. Chemically, it is a derivative of both unsaturated diketones and phenols. For this reason, this molecule may also exist in the form of a tautomeric variant remaining in equilibrium with the structure of THC (Fig 1).

Emerging evidence demonstrated that THC possesses potential applications in curcumin-induced pharmacological effects and less toxic adverse outcomes compared to curcumin in cancer treatment [4–6]. Intriguingly, the data revealed that numerous clinical trials have addressed the benefits, but its detailed mechanism of action remains to be elucidated. Despite its benefits, THC has been reported to be unstable in the gastrointestinal tract and has poor solubility, leading to low systemic bioavailability in physiological circumstances [7, 8]. Currently, several nanoformulations of THC have been developed to improve its solubility such as liposomes, microspheres, osmotic pumps, nanoemulsions, and solid dispersion systems. However, many of these existing formulations may not fully preserve the pharmacological activity of THC, thus minimising its potential toxicity, targeted delivery and extended shelf-life [9–12]. Hence, a carrier method that is both effective and safe is important to improve water-soluble delivery systems for use in clinical applications.

β-cyclodextrin (βCD), a natural cyclic bucket-shaped oligosaccharide expressing a hydrophobic inner cavity and an external hydrophilic surface, was selected among various carriers as a receptacle for THC delivery applications [9]. The unique characteristics and abilities of the nonpolar cavity of βCD enable it to readily replace the poorly soluble guest molecules via host-guest interactions, particularly in an energetically unfavoured state, and form an inclusion complex for the establishment of a controlled release system underlying distinct stimulus-response mechanisms [10, 11]. Several nanoparticles developed with βCD as a carrier have been intricately crafted for targeted application in cancer treatment. For instance, Paclitaxel, Doxorubicin and small interfering RNA (siRNA) targeting oncogenes [13, 14].

This study aims to evaluate the *in vitro* and *in vivo* anticancer properties of native THC and its complexation with βCD as a drug delivery system using colorectal cancer (CRC) as a model disease. The research was also supported by *in silico* analysis using the SwissTargetPrediction server. An inclusion complex formulation that improves solubility and stability could therefore be used and extrapolated for various chemotherapeutic applications.

## Materials and methods

### *In silico* analysis of THC

*In silico* analyses were performed using the available Swiss server (SwissTargetPrediction). The THC molecule was read according to the Smiles code.

### Preparation of water-soluble inclusion complex

THC (cat. no. FT28087, > 96% purity, MW 372.4 g/mol) was obtained from Carbosynth Ltd, UK and βCD (cat. no. C8510; > 97% purity, MW 1135.0 g/mol) was purchased from Sigma-Aldrich (St. Louis, MO, USA). βCD and THC were prepared in a 2:1 M ratio of individual dispersion in deionised water (dH_2_O) and 99.7 v/v % anhydrous ethanol, respectively. Both solutions in a closed dark glass container were gently agitated and heated at 60°C for 4 h without a cap to allow the ethanol to evaporate. The reaction mixture was cooled to ambient temperature and stirred for another 5 h, then stored overnight at 4°C. The solution was then centrifuged at 1,000x*g* for 15 minutes and the supernatant was recovered by 0.45 μm vacuum filtration followed by pressure distillation. Then, the filtrate was dried in a 60°C oven for 48 h. The highly water-soluble βCD-THC inclusion complex was stored at room temperature for further characterisation [15].

### Spectroscopic studies

The samples of βCD, pure THC, and βCD-THC inclusion complex were subjected to initial physiochemical characterisation by spectroscopy. Absorption spectra were recorded in the range from 250 to 400 nm using the UviLine 9400 spectrophotometer (Secomam, France) in their respective solvent systems as a blank to determine the wavelength of maximum absorption (λmax).

### FTIR spectrum

FTIR spectra were performed using Nicolet™ Summit Spectrometer (Thermo Fisher Scientific, Inc., Waltham, MA, USA). Each sample was placed on the spectroscopic grade potassium bromide (KBr) disc container, and data was acquired for the scanning range using OMNIC software from 4,000 to 650 cm^‑1^ at ambient temperature in the transmission mode.

### DSC analysis

βCD, pure THC, and βCD-THC inclusion complex samples were sent to the Institute of Nanoscience and Nanotechnology, University Putra Malaysia (UPM) for DSC analysis. The DSC measurement was analysed by a DSC822e differential scanning calorimeter (Mettler Toledo, Columbus, Ohio, US). Approximately 5 mg of each sample was scanned under 50 mL/minute ultrahigh-purity nitrogen gas purging and heated in a completely sealed aluminium pan covered with an aluminium lid at a heat ramping rate of 10˚C/minute with a temperature range of 35 to 300˚C.

### SEM imaging

Samples were sent to the Microscopy Unit, Institute of Bioscience, UPM for SEM imaging. All samples in powder form were mounted on a carbon tape and coated with a gold film. The surface morphology of samples was directly observed without fixation using a JSM-IT100 InTouchScope™ scanning electron microscope (JEOL SEM technologies, Peabody, MA, USA) at 20 kV.

### Drug content analysis

βCD-THC inclusion complex (10mg) was diluted in ethanol and gently agitated for 24 h at room temperature in the dark to determine THC content for loading estimation. To dissociate the captured THC from βCD, the clear supernatant containing THC-extracted ethanol solution was centrifuged at 16,000x*g* for 10 minutes. Absorbance was measured at 284 nm using UviLine 9400 spectrophotometer (Secomam, France). Meanwhile, a seven-point calibration curve of equivalent pure THC was prepared under identical conditions. THC quantification was determined using the following equation: Inclusion complex formation efficiency (%) = mass of complexed THC/total THC added initially x 100 [16].

### Solubility of the βCD-THC inclusion complex

Pure THC (42 μg) and βCD-THC inclusion complex (300 μg) were dissolved in dH_2_O separately and vortexed for 1 minute. The samples were centrifuged at 1,600x*g* at 25˚C for 5 minutes, followed by a filtration process through a 0.45 μm filter membrane to remove insoluble particles. An aliquot was taken for spectrophotometric analysis using UviLine 9400 spectrophotometer (Secomam, France). All experiments were carried out in independent triplicates [17].

### Drug release study

The dissolution of the βCD-THC inclusion complex was employed to evaluate the THC release profile. The study was performed using a size-restricted dialysis tubing procedure to determine the THC release from known amounts of complex, respectively. Dialysis bags (MWCO 8–10 kDa, Sigma, Germany) were filled with a known amount of complex along with 0.5% sodium lauryl sulfate (SLS) in 50 mL of dissolution medium at pH 1.5 for 2 h. The thermostated water bath system was controlled at 37°C with an agitation of 150 rpm. After 2 h, the previous buffer was replaced by pH 6.8 buffer for another 4 h. Aliquots were taken from the receiver part at the given time interval and refilled with an equal volume of fresh dissolution medium. The withdrawn sample was filtered through a 0.45 μm pore size syringe filter and subjected to quantitative analysis by UviLine 9400 spectrophotometer (Secomam, France) at 284 nm. All measurements were performed in triplicates [18].

### Cell lines and culture conditions

Human CRC cell lines, HCT116 (*ATCC*® CCL-247^™^) and SW480 (*ATCC*® CCL-228™) and normal human dermal fibroblast (NHDF) (*ATCC*® PCS-201-012™) were purchased from American Type Culture Collection (ATCC; Manassas, VA, USA). The cells were cultured as monolayers in Dulbecco’s Modified Eagle’s Medium (DMEM; Gibco, Thermo Fisher Scientific, Inc., USA) supplemented with 10.0% (v/v) fetal bovine serum (FBS; Gibco, Thermo Fisher Scientific, Inc., USA), and antibiotics (100 U/mL penicillin and streptomycin; Gibco, Thermo Fisher Scientific, Inc., USA). All cultures were maintained in an incubator at 37° C with a 5% CO_2_ atmosphere and 95% humidity level.

### Cytotoxicity assay

The anti-cancer activity was assessed using a cell proliferation assay that measured MTT 3-(4,5-dimethylthiazol-2-yl)-2,5-diphenyl tetrazolium bromide dye uptake and metabolism. All compound samples were prepared at a concentration ranging from 2.7 to 268.5 μM. An equal number of cells (1.0 x 10^4^ cells/well) were incubated at 37˚C for 24 h after the addition of samples while the negative control (medium only) and positive control (1 μM camptothecin) were also included. The MTT dye reagent (0.5 mg/mL) was added to each well and the plates were incubated in the dark at 37°C with 5% CO_2_ for 2 h. The resultant purple formazan crystals were solubilised in 100 μl of DMSO, followed by monitoring the absorbance intensity at 570 nm using a FLUOstar Omega microplate reader (BMG Labtech, Ortenberg, Germany) against a DMSO blank. All assays were performed in triplicates, and the data of relative cell viability was normalised as a percentage relative to the corresponding vehicle-treated controls [19, 20].

### Migration assay

Anti-migration effects were determined using the wound healing assay. An equal number of cells (1 x 10^5^ cells/well) were seeded and cultured at 37°C with 5% CO_2_ for 24 h. The growth medium was changed to a medium containing 5 μg/mL of mitomycin-C (Sigma-Aldrich, St. Louis, MO, USA) and further incubated at 37°C for 2 h to halt cell proliferation. Scratch wounds of equal size were introduced into the monolayer by a sterile yellow pipette tip and cell debris generated from the scratch was washed twice with 1x phosphate-buffered saline (PBS). Medium along with IC_20_ of samples were used to treat cells. Microscopic images describing the degree of wound closure were documented at 0 h and 24 h post-wounding using the Nikon Eclipse TS100 inverted fluorescence microscope (Nikon Instruments, Fujisawa, Japan) and the distances between the wound edges were analysed using ImageJ v1.43 analysis software (NIH, Bethesda, MD, USA). All experiments were performed in triplicates (n = 3) [13, 21].

### Transwell invasion assay

Cell invasion capacity was examined by measuring the number of cells transmigrating through a layer of the extracellular matrix, Matrigel. The 24-well transparent PET membrane inserts with 8.0 μM pore sizes were coated with 40 μL of 1 mg/mL Matrigel (BD Biosciences, USA) and incubated for 2 h at 37°C. An equal number of cells (1.0 × 10^5^ cells/well) were grown as monolayers on the upper insert for 24h. The cells were starved in serum-free medium along with samples at IC_20_ concentrations whilst medium with 10% (v/v) FBS was added to the lower insert as a chemo-attractant. Invasion was allowed to occur for 24 h at 37°C with 5% CO_2_. Invading cells on the underside of the membrane were fixed and stained with 1% (w/v) crystal violet (Sigma, USA). The absorbance reading was taken after cells were eluted with acetic acid. All experiments were performed in triplicates (n = 3) [22].

### Annexin V-FITC/PI staining

Cells were seeded with a density of 1 x 10^5^ cells/well and incubated overnight at 37°C in 5% CO_2_. In brief, cells were treated with samples using IC_50,_ followed by a washing step using cold 1x PBS twice. A binding buffer suspension of 500 μL was added to the collected cells. Apoptosis was detected by initially staining the cells with 0.1% (v/v) Annexin V-FITC and 100 mg/mL of PI (cat. no. CA1020-50, Solarbio Beijing, China). The stained cell suspensions in each tube were left at room temperature for 15 minutes in the dark, and flow cytometry (BD FACS Caliber instrument, BD Biosciences, San Jose, CA, USA) (Ex = 488 nm, Em = 530 nm) was used to measure the number of cells in each tube [23].

### PARP cleavage assay

Cells (1.0 x 10^7^/mL) were treated with samples using IC_50_ and total proteins were extracted using the ProteinExt® mammalian nuclear and cytoplasmic protein extraction kit (TransGen Biotech Co., Ltd., Beijing, China) according to the manufacturer’s protocol. Protein concentration was quantified and normalised using the Quick Start Bradford Protein Assay Kit 2 (Bio-Rad, Irvine, CA, USA). Fractionation was done using SDS-PAGE and transferred onto a polyvinylidene fluoride (PVDF) membrane (Thermo Fisher, Waltham, MA, USA) for western blot analysis using a wet transfer system (Bio-Rad, Irvine, CA, USA). The membrane was incubated with primary antibody PARP 1 (1:1000) (cat. no. E-AB-16025, Elabscience Biotechnology Inc., USA) and beta-actin (1:2000) (cat. no. E-AB-20058, Elabscience Biotechnology Inc., Houston, TX, USA) was then introduced into the blocking solution at concentrations of 10.0 ng/mL, followed by an additional 1 h incubation at room temperature with gentle shaking. Following this, the solution was discarded, and the membrane underwent washing with 1× TBST. A brief 5-minute incubation with gentle agitation was followed by discarding the washing solution. This washing process was repeated twice before introducing 10.0 mL of secondary HRP-conjugated goat anti-rabbit IgG (cat. No. E-AB-1003, Elabscience Biotechnology Inc., Houston, TX, USA) diluted at 1:5000 in 1% BSA. The TMB substrate was added to the surface of the membrane and specific protein bands were analysed using a GS-800 Calibrated Imaging Densitometer (Bio-Rad, Irvine, CA, USA). Apoptosis was represented by the cleavage of 116-kDa PARP into an 89-kDa fragment [24].

### Animal care and husbandry of female BALB/c nude mice

Six groups (n = 5) of 4-week-old female athymic BALB/c nude mice (Nomura, Thailand), with a body weight range of 16–20 g, were procured from the Animal Resource Unit, Faculty of Medicine, UPM. These mice were housed in a specific-pathogen-free facility (SPF) at the Comparative Medicine and Technology Unit (COMeT), UPM. The mice were accommodated in cages with corn cob bedding, ensuring *ad libitum* access to food and water. The animal housing facility maintained a well-ventilated environment at a temperature of 25°C. Stringent adherence to ethical guidelines was followed, specifically the “Animal Research: Reporting of *in vivo* experiments (ARRIVE)” guidelines, to ensure proper animal care and husbandry practices. A period of 2 weeks was allocated for the acclimatization of all mice before the start of tumour inoculation.

### *In vivo* efficacy of inclusion complex formulations

Animal experiments were conducted in compliance with the “Guide for the Care and Use of Laboratory Animals” (National Research Council, 2011) guideline under Institutional Animal Care and Use Committee (IACUC) ethics approval (UPM/IACUC/AUP-R049/2022). A set of studies was conducted to evaluate the therapeutic effects on tumour xenografts simultaneously. Tumour xenografts were induced by subcutaneously inoculating them with 1 × 10^7^ cancer cells suspended in PBS and BD Matrigel Matrix HC (1:1) (100 μL/mice) into the neck region using 25-gauge needles. All formulations were prepared in PBS and administered intraperitoneally (i.p.) when the tumour load reached a threshold of 100 mm^3^. The following treatment groups (n = 5) were assigned: (i) placebo (0.9% NaCl), (ii) 2 mg/kg camptothecin, (iii) 44mg/kg THC and (iv) 312mg/kg βCD-THC. A total of 7 drug treatments were administered biweekly with a 2-day interval, including the tumour induction period. The xenograft tumour size was measured every 2 days with a digital calliper, and the volume was calculated according to the formula (W (2) x L)/2, where L and W represent the length and width, respectively [25]. All mice were euthanised by CO_2_ asphyxiation at the end of the study, day 21. Tumours were harvested, rinsed with normal saline and fixed in 10% neutral buffer saline.

### Collection of whole blood, tumour, kidney, and liver samples

Before sample collection, mice were first anaesthetised. Initially, the injection site was sterilised with 70% (v/v) ethanol. Subsequently, anaesthesia was administered via an i.p. injection using a 30-gauge needle, with a cocktail containing 93.33 mg/kg of ketamine and 1.33 mg/kg of xylazine for every 20.0 g of mice body weight. The mice were allowed to undergo a numbing onset lasting 5–10 minutes, and their consciousness was assessed using a toe pinch test. Whole blood was obtained through cardiac puncture and collected in clean microcentrifuge tubes. The blood was allowed to clot at room temperature for 30 minutes. Then, serum samples were harvested from clotted blood by centrifugation at 1,000xg for 10 minutes. The upper layer of serum was collected, aliquoted into centrifuge tubes and stored at -20° C until further use.

A small piece of tissue was cut on dry ice with a scalpel and rinsed in ice-cold PBS (0.01M, pH = 7.4) to thoroughly remove excess blood. Tissue pieces were weighed and then homogenised in PBS (tissue weight (g): PBS (mL) volume = 1:9) with a glass homogenizer on ice. Subsequently, this small tissue piece was minced into even smaller fragments using the same scalpel. Subsequently, 0.05–0.5 g of tissue was placed into a 1.5 mL homogenizer tube on wet ice, and the homogenizer tube was filled with lysis buffer. The sample was homogenised in the tube for 90 seconds and placed on ice. The homogenates were then centrifuged for 10 minutes at 5000×g at 4°C to get the supernatant and kept on ice until use. Protein concentration was determined using the Bradford assay.

### CEA levels detection

The enzyme-linked immunosorbent assay (ELISA) kit (cat. no. E-EL-M0232, Elabscience Biotechnology Inc., Houston, TX, USA) was used for the quantitative determination of cancer antigen serum levels following the manufacturer’s protocol. Briefly, samples were added with monoclonal HRP-conjugated anti-CEA IgG after being immobilised with goat antibody at room temperature. After 2 hours, the bound HRP conjugate was detected by employing 3,3’,5,5’- tetramethylbenzidine (TMB) solution in the dark at room temperature. The peroxidase reaction was halted by adding HCl stop solution after 30 minutes, followed by the measurement of optical densities at 450 nm using a spectrophotometer. A linear standard concentration range (0 ng/ml to 120 ng/ml) was used to correlate absorbance values to concentration values.

### CYP1A2 inhibition response

Mice CYP1A2 (cat. no. E-EL-M2643, Elabscience Biotechnology Inc., Houston, TX, USA) was used on mice liver microsomes. Briefly, a mixture consisting of 1 mg/ml microsomal protein, 0.1 M phosphate buffer (pH 7.4), 1 mM NADPH, and CYP isoform-specific individual substrates were incubated until reaching a final volume of 200 ml. The substrates were dissolved and serially diluted with acetonitrile to the required concentrations. After 10 minutes of incubation, the reactions were terminated by adding 200 ml of cold acetonitrile containing carbamazepine (50 nM), followed by agitation with a vortex mixer. Samples for each enzyme assay were centrifuged (1000 xg, 20 minutes, 4°C), and the supernatants of the reaction samples were analysed by ELISA using the Sandwich-ELISA principle.

### H&E staining

H&E staining was used to examine the histopathology of tissue sections derived from liver, kidney, and tumour specimens. The samples underwent a comprehensive histological processing sequence. Initially, they were fixed in 10% (v/v) neutral buffered formalin, followed by dehydration in a series of graded ethanol solutions and subsequent clearing in xylene, facilitating optimal paraffin embedding. Post-embedding, thin sections, 4–5 m thick, were obtained using a microtome and delicately floated onto glass slides. The staining process involved the deparaffinization of slides using xylene and subsequent rehydration through descending concentrations of ethanol. Hematoxylin staining was then employed to visualize nuclei, with the slides immersed in the dye for 5 minutes. Following hematoxylin staining, the slides were rinsed under running water to eliminate excess stains. Eosin served as a counterstain, imparting a pink tint to the cytoplasm and extracellular structures. After staining, slides were dehydrated using an ethanol series, cleaned in xylene, and covered with mounting media. The produced slides were subjected to histological examination under a light microscope, which revealed clear visualizations of tissue architecture, pathogenic abnormalities, and cellular morphology.

### Immunohistochemical analysis (IHC) of tumour biopsies

Formalin-fixed paraffin-embedded (FFPE) tumour biopsies were subjected to paraffin removal using xylol and rehydration in a graded alcohol series (Cell Signalling, USA). Endogenous peroxidase activity was blocked by incubating the sections in a 3% H_2_O_2_ solution in methanol at room temperature for 10 minutes, followed by rinsing in 300 ml of PBS for two changes, each taking 5 minutes. Antigen retrieval was performed to unmask the antigenic epitope using a citrate buffer method. IHC was performed using antibodies specific for NFκB p65 (1:200) (cat. no. E-AB-67474, Elabscience Biotechnology Inc., USA) and 0.5% bovine serum albumin in PBS for 1 h. The slides were washed in 300 ml PBS for two changes, each taking 5 minutes. Subsequently, 100 μl (1:10000) biotinylated secondary antibody (Polyperoxidase-anti-Rabbit IgG) was applied and incubated in a humidified chamber at room temperature for 20 minutes. The slides were washed in 300 ml PBS for two changes, each lasting 5 minutes. Signal detection was done using DAB (100 μl) for 5 minutes. The slides were washed in 300 ml PBS for three changes, each taking 2 minutes. Counterstaining was performed using hematoxylin (Sigma-Aldrich, MO, USA) by immersing the samples in hematoxylin for 1–2 minutes. The slides were rinsed in running tap water for 15 minutes. The tissue slides were dehydrated through four changes of alcohol (95%, 95%, 100%, and 100%), each lasting 5 minutes. The sections were embedded with dibutyl phthalate xylene (DPX) mounting medium and the cover was slipped using a mounting solution (Permount). The colour of the antibody staining in tissue sections was observed using an inverted microscope Nikon Eclipse TS 100 (Nikon Instruments, Japan) and quantified using the Nikon NIS-BR Element software (Nikon Instruments, Japan).

### Statistical analysis

All data were expressed as mean ± standard deviation (S.D) using Microsoft Excel. The statistical significance analysis was calculated using Student’s two-tailed t-test, and data significance thresholds were shown as *p < 0.05, **p < 0.01, ***p < 0.001, ****p < 0.0001 and *****p < 0.00001.

## Results and discussion

### *In silico* results of THC

**Fig 2:**
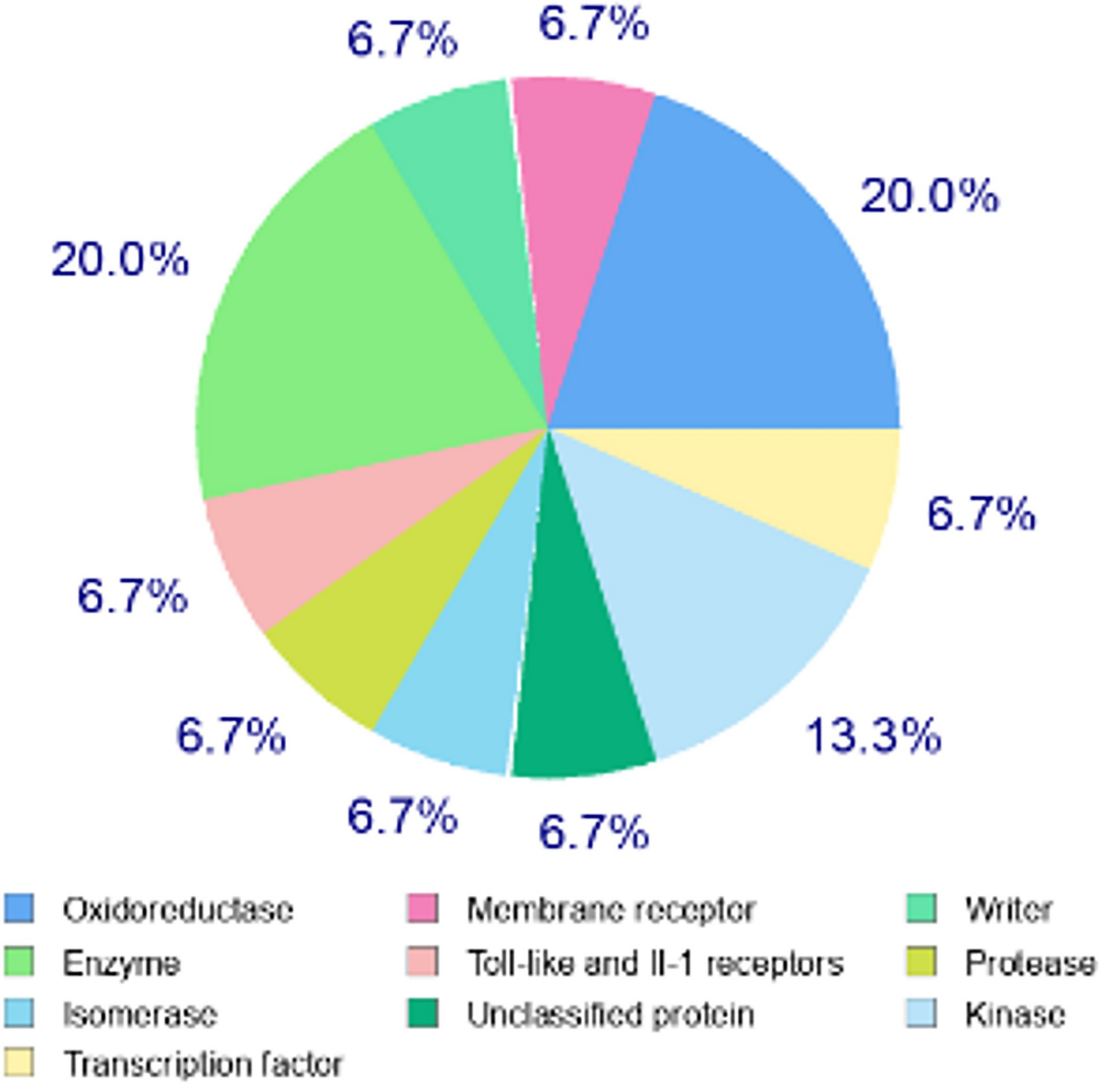
Target classes of THC.

Using the SwissTargetPrediction server, molecular targets of THC were determined, which indicated the probability of activity in relation to histone acetyltransferase p300, DNA topoisomerase II alpha, prostaglandin E synthase, monoamine oxidase A, beta-amyloid A4 protein, toll-like receptor (TLR7/TLR9), inhibitor of NF-kappa-B kinase (IKK), beta-secretase 1, nuclear factor erythroid 2-related factor 2, arachidonate 5-lipoxygenase. These preliminary analyses confirmed literature reports indicating the high potential of anticancer and anti-inflammatory activity of THC. The molecular targets indicated for THC are presented in Fig 2.

### Absorption characterisation

**Fig 3:**
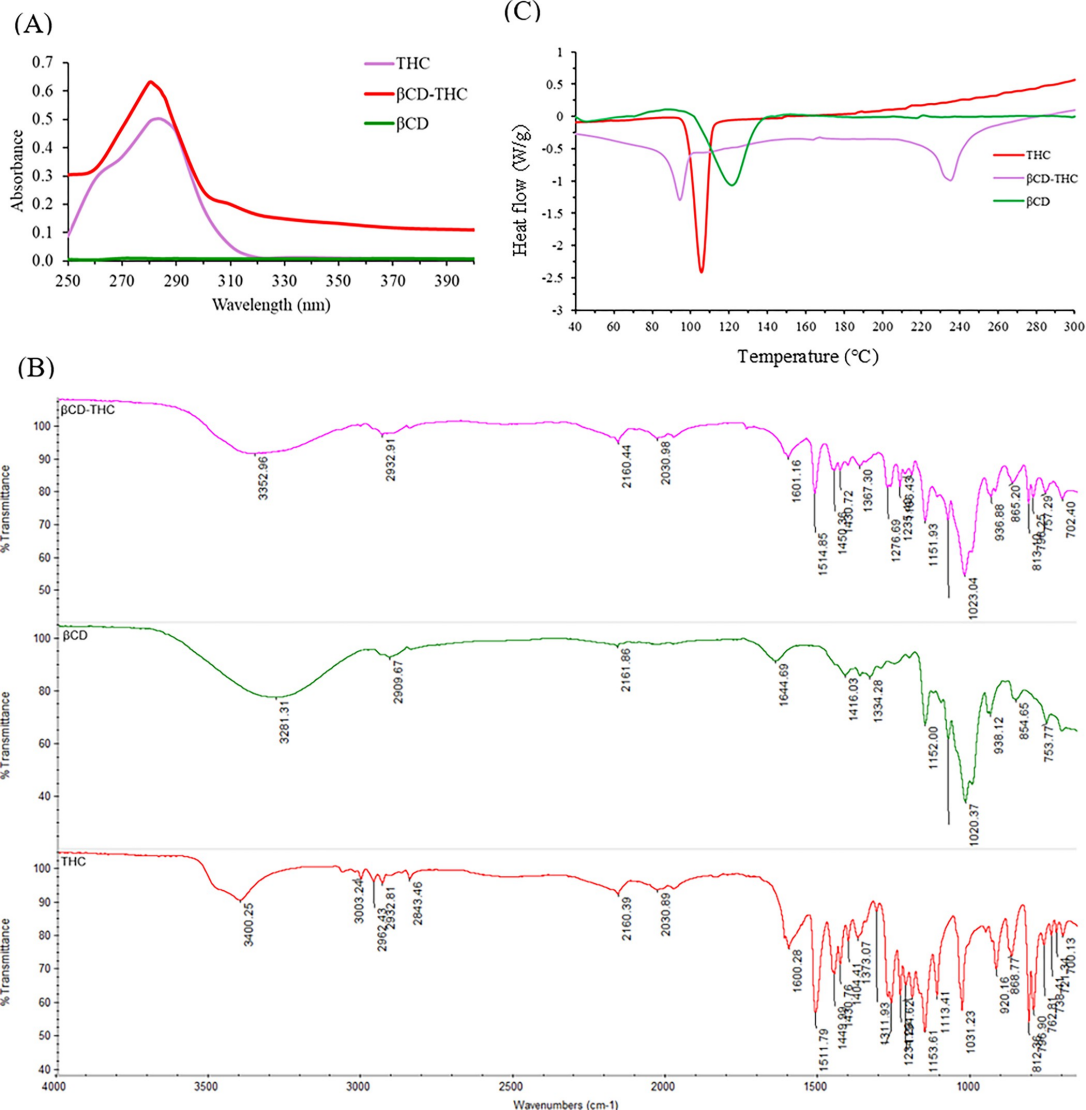
Initial characterisations of the βCD-THC inclusion complex were compared with pure THC and βCD. (A) Absorption spectra (250–400 nm). (B) FTIR spectra (4000−1000 cm−1). (C) DSC exothermic curves.

As shown in Fig 3A, UV spectra were used to confirm the successfully functionalised inclusion complex. The intense absorption band of THC was observed at 284 nm, while βCD showed no significant maximum absorption peak in this region. The absorption spectrum shape of THC was similar to the βCD-THC inclusion complex, and the noticeable increase in the absorption intensity of the βCD-THC inclusion complex and its λ_max_ shift to 280 nm was rationalised as an indicator of inclusion complex formation [26]. Generally, the absorbance of the guest molecule would be enhanced upon entrapment in the βCD cavity due to the increase in molar absorption coefficient during the complexation [27]. These results indicate that the inclusion complex was formed.

Poor water solubility and low bioavailability, which severely constrained THC’s chemotherapeutic efficacy were important challenges that could be resolved with this complexation. THC was complexed with βCD at a 2:1 molar ratio to accomplish this. According to the steric factors of forming inclusion and demonstrating the maximum binding constant during the complexation, researchers have shown that the aromatic ring of THC was ideal for entrapment within the inner cavity of βCD at this molar ratio [28]. Due to its aromatic rings’ hydroxyl and ether groups, THC extremities are characterised by a strong peak at 284 nm [29]. The spectrum of the βCD-THC inclusion complex in particular exhibited two distinct peaks at 280 nm and 284 nm, as well as the displacement of the peak centre. This demonstrated the presence of THC in the finished product and the partial shielding of excitable electrons and chromophores of functionalised βCD [30].

### Chemical component analysis

An FTIR spectrum was presented in Fig 3B to further confirm that THC was successfully encapsulated in the inner cavity of βCD. The specific IR absorption bands of THC (red line) were exhibited at 3400 cm^-1^ (phenolic O-H), 1600 cm^-1^(C = O), 1512 cm^-1^ (C = C benzene ring) and 812 cm^-1^ (C-O-C glucose unit). The spectrum of βCD (green line) produced peaks at 3281 cm-1 (O-H), 2910 cm^-1^ (C-H), 1152 cm^-1^ (C-O), 1,020 cm^-1^ (C-O-C glucose units) and 855 cm^-1^ (C-O-C rings of βCD). Meanwhile, all the sharp peaks of βCD-THC revealed that the spectrum belonged to βCD while only a few characteristic peaks of THC appeared, as βCD-THC inclusion complex (pink line) peaks were shifted to a higher to lower wavelengths, such as at 3281 to 3352 cm^-1^, 1644 to 1601 cm^-1^ and 855 to 865 cm^-1^. The multiple OH functional groups of βCD corresponded to the OH stretching band of the βCD-THC inclusion complex. The results also showed that the interaction of the hydrogen bond and van der Waals forces during the complexation was caused by the microenvironment [31, 32]. This data confirmed the complexation of THC with βCD. All these findings offer strong proof that an inclusion complex had formed. Except for a few THC peaks, the data showed that all other peaks belonging to βCD emerged and moved to higher or lower frequencies during complexation, which was explained as being indicative of complex formation [33].

### Thermal properties of the THC inclusion complex formulation

The thermograms obtained for THC, βCD and βCD-THC are presented in Fig 3C. The decomposition of THC resulted in a sharp endothermic peak at 105.4°C. The thermal profile of βCD-THC was enormously reduced compared to THC, with a small peak of around 94.1°C. A shift from the peak that corresponded to βCD was visible, and the melting peak was nearly non-existent. This result implies that THC was wrapped into the inner cavity of βCD. This complex also promoted greater thermal protection of THC, causing degradation of ions around 235°C. The results showed that the combined characteristics of THC and βCD had undergone physical changes at the molecular level. This is explained by the fact that the DSC curve of the βCD-THC inclusion complex exhibits βCD-like characteristics, indicating a strong interaction between βCD and THC molecules in mixed states and the development of the inclusion complex, which is supported by the occurrence of expanding peaks. The DSC curve indicated that the physical changes of amorphization and melting point were the primary causes towards the disappearance of the endothermic peak. The displaced peaks were discovered to be consistent across numerous experiments [34, 35]. In addition, it also showed the shift of the endothermic peak to different temperatures, indicating a change in the crystal lattice, melting, boiling or sublimation points, thereby confirming the presence of this new peak, which is evident for the formation of an inclusion complex [36].

### Morphological identification

**Fig 4:**
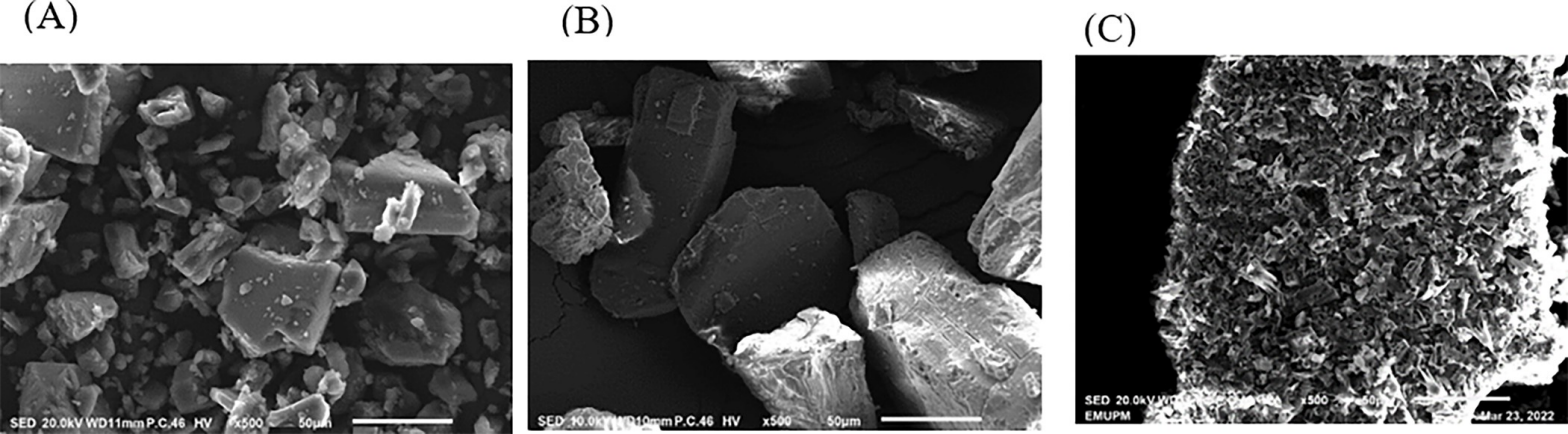
SEM photographs. (A) THC, (B) βCD and (C) βCD-THC inclusion complex was observed under 500x magnification.

A qualitative method was used to study the overall bulk surface morphology of THC, βCD and βCD-THC inclusion complexes by SEM (Fig 4). As seen in Fig 4A, THC appeared as irregular-shaped crystal particles while the morphology of βCD (Fig 4B) was presented in a polyhedral form with crystalline flake-like structures. These features were not comparable with the βCD-THC inclusion complex as seen in the parallelogram (Fig 4C) since the original morphology of both components had disappeared. By displaying a mixture of big and small irregular-shaped clumps, the βCD-THC inclusion complex was identified as a morphological combination of both parent chemicals. This morphological modification indicated that an inclusion complex had successfully formed. The SEM findings were in good accord with a few publications [33, 37], which attributed this to pure THC being encapsulated within the inner chamber of the βCD as tiny aggregates in a flake-like form. The uniform crystal particle shape adhered to the surface of the βCD-THC inclusion complex, demonstrating the apparent interaction between βCD and THC in a solid state [38].

### Assessment of solubility and THC release behaviours of the inclusion complex

The amount of THC in the inclusion complex was 10 μg/ml following 67.21% inclusion complex efficiency through absorbance measurements at 284 nm using a UV spectrophotometer. The higher water solubility properties of the βCD-THC inclusion complex increased from 1.3% to 84.5% compared to THC. A clear solution was obtained for the βCD-THC inclusion complex in an aqueous solution, while THC was presented with undissolved particles aggregated at the bottom of vials. Therefore, these solubility results were satisfactory for a water-soluble inclusion complex and should demonstrate an increase in bioavailability [27].

**Fig 5:**
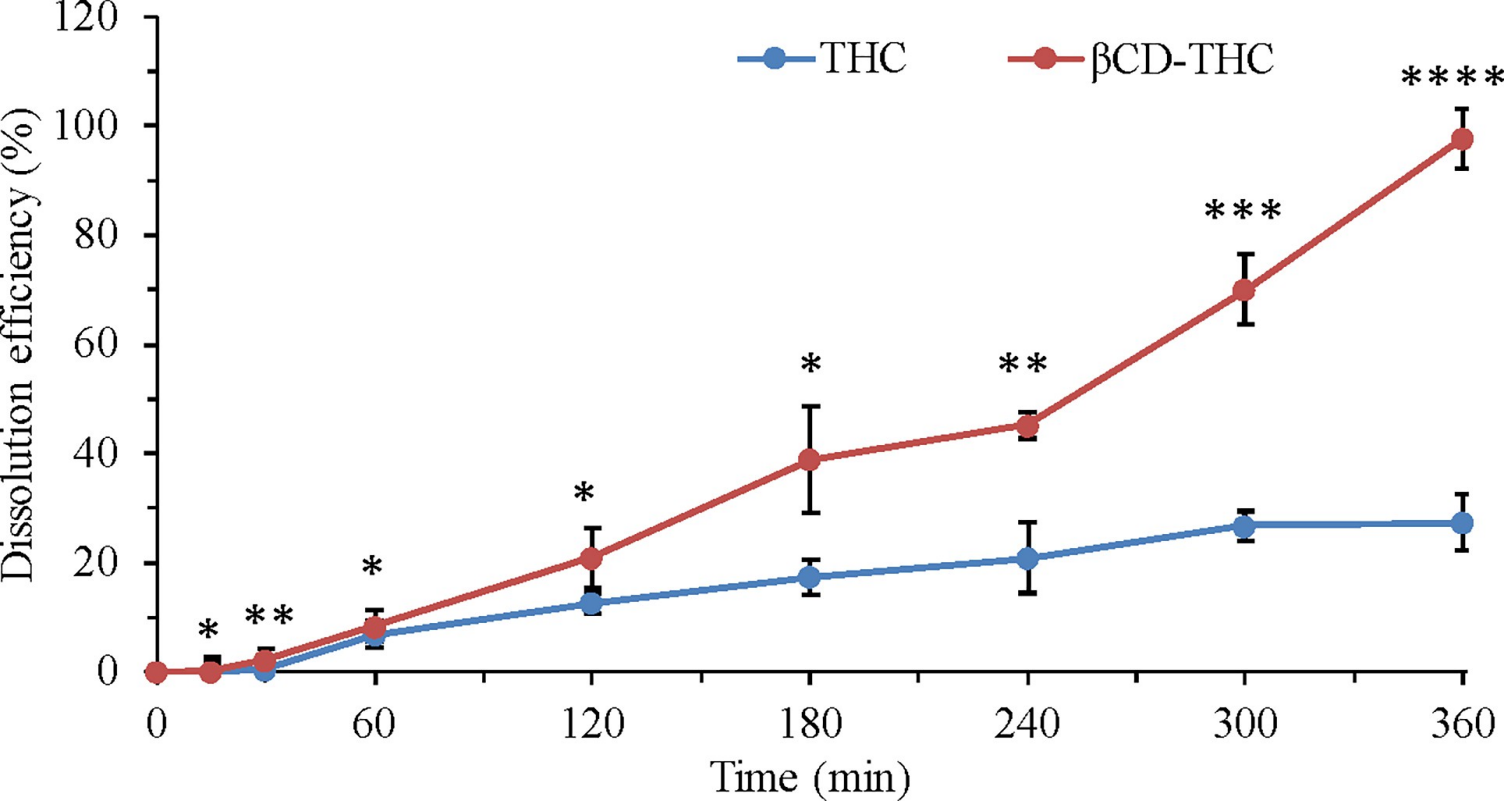
Release profile of THC from the βCD-THC inclusion complex versus pure THC, βCD-THC = percentage of THC concentration in the βCD-THC 2:1 complex. All results were presented as mean ± SD., *p < 0.05, **p < 0.01, ***p < 0.001 and ****p < 0.0001.

Fig 5 illustrates the cumulative dissolution profile of THC and βCD-THC inclusion complexes. As seen in Fig 5, the βCD-THC inclusion complex showed a remarkable increase in the dissolution rate compared to that of THC. It took almost 120 minutes for 12.70% of the drug to be released from THC compared with the release of 21.05% from the βCD-THC inclusion complex. At 360 minutes, 27.39% and 97.90% were released from THC and βCD-THC inclusion complexes, respectively. In this case, a near-complete disintegration of βCD-THC inclusion complexes was observed.

This was attributed to βCD exhibiting the capability to form a stable inclusion complex with THC at the molecular level, primarily driven by hydrophobic interactions as the THC molecule inserts into the βCD cavity. Consequently, the formulation ensures that most of the drug is released upon reaching the colon, with a lag time of five hours deemed sufficient to ensure exclusive drug release within this specific gastrointestinal region [17, 18]. These data showed a delayed release in the gastric fluid and postulated that the inclusion complex could act adequately as a colon-delivered formulation [18, 39]. Therefore, these results show an improvement in dissolution kinetics in comparison to THC alone, confirming its superiority in solubility and drug wettability in buffers at physiological pH conditions [40].

### *In vitro* determination of growth inhibition following treatment with THC inclusion complex

**Fig 6:**
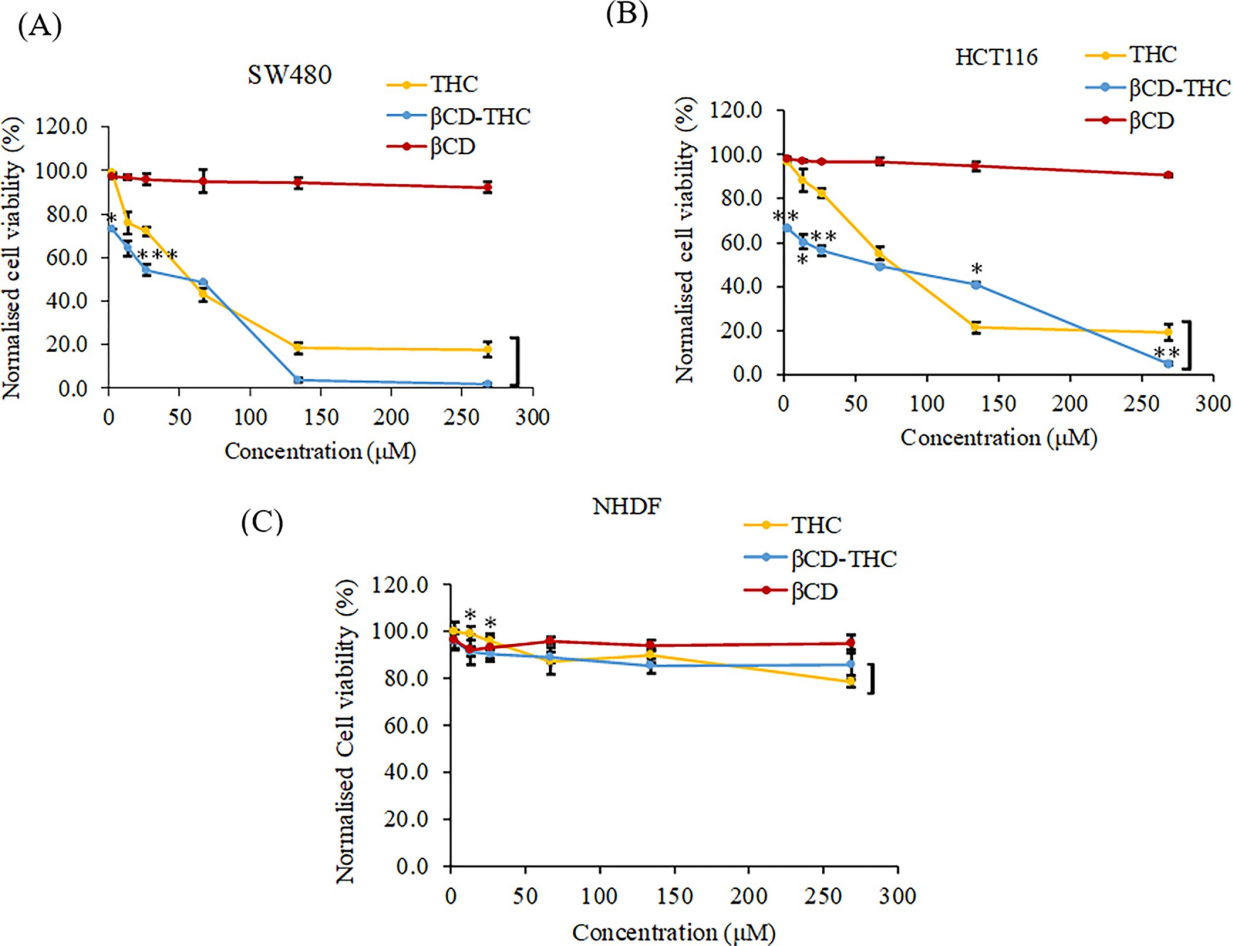
Normalised cell viability comparison of THC, βCD and βCD-THC inclusion complexes treated for 24 h on (A) SW480, (B) HCT116 and (C) NHDF cells. Each set of data shown is presented as mean ± S.D. from triplicate experiments, where significant differences between THC and βCD-THC are denoted as *p < 0.05, **p < 0.01 and ***p < 0.001.

To evaluate the inhibitory effect of the βCD-THC inclusion complex against cancer and normal cell lines, the cytotoxicity activity of the different concentrations of samples was analysed for 24 h using the MTT method. Camptothecin, a plant-derived compound, was used as a positive control as it has equivalent properties to THC, but with poor aqueous solubility that requires DMSO to dissolve (Fig 6). Subsequently, a dose-dependent reduction of cancer cell proliferation was observed with THC and βCD-THC inclusion complex, but not with βCD alone. Based on these measurements, the IC_50_ doses of THC were 57.2 μM and 77.3 μM for SW480 and HC116 cells, respectively (Fig 6A and 6B). The βCD-THC inclusion complex exhibited a therapeutic IC_50_ dose of 56.2 μM and 62.7 μM respectively, indicating an increase in cytotoxic efficacy in the tested cell lines. The selectivity index (SI) value = (IC_50_ normal cell / IC_50_ cancer cell line) was calculated to determine the cytotoxicity of samples against non-malignant cell lines [41]. The toxicity of THC and βCD-THC inclusion complex was not apparent on NHDF normal cell lines (Fig 6C), which resulted in more than 78.6% (SI value > 10 for SW480 cells and SI value > 3 for HCT116 cells) and 85.3% (SI value > 10 for both SW480 and HCT116 cells), respectively. Therefore, the βCD-THC inclusion complex was deemed a potential drug candidate that causes less harm when applied to humans and warrants further investigation.

### Cell motility measurements via migration and invasion assays

**Fig 7:**
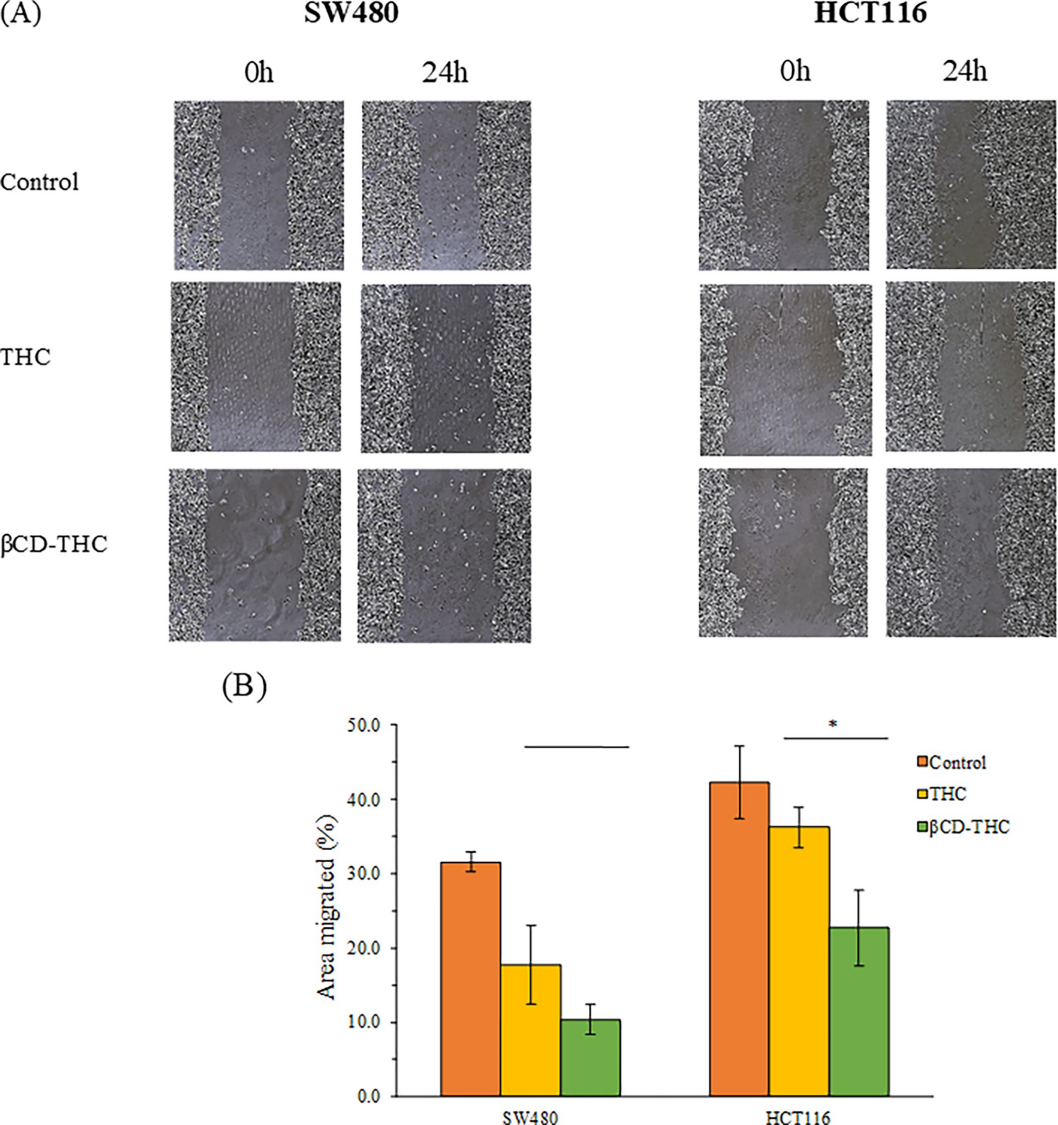
The cell migration effects of THC and βCD-THC inclusion complex on SW480 and HCT116 cells. (A) Representative images at 100x total magnification of scratch assays. (B) Bar charts present the percentage of the distance between the boundaries of the migrating cells from three replicates. Statistically significant differences in comparison between THC and βCD-THC groups are marked with (*), denoting *p* < 0.05.

A scratch assay was performed to investigate the cell migratory inhibitory effects on CRC cell lines. The image of the wound areas was evaluated as shown in Fig 7A. The difference in migration for 24 h of THC underlying HCT116 cells was statistically significant (*p* < 0.05) in comparison with the βCD-THC inclusion complex, which was 22.73% and 36.20%, as seen in Fig 7B. For SW480 cells, the percentage of cell migration area of THC was shown to be 17.75%, while the βCD-THC inclusion complex recorded a migration of 10.40% (Fig 7B).

**Fig 8:**
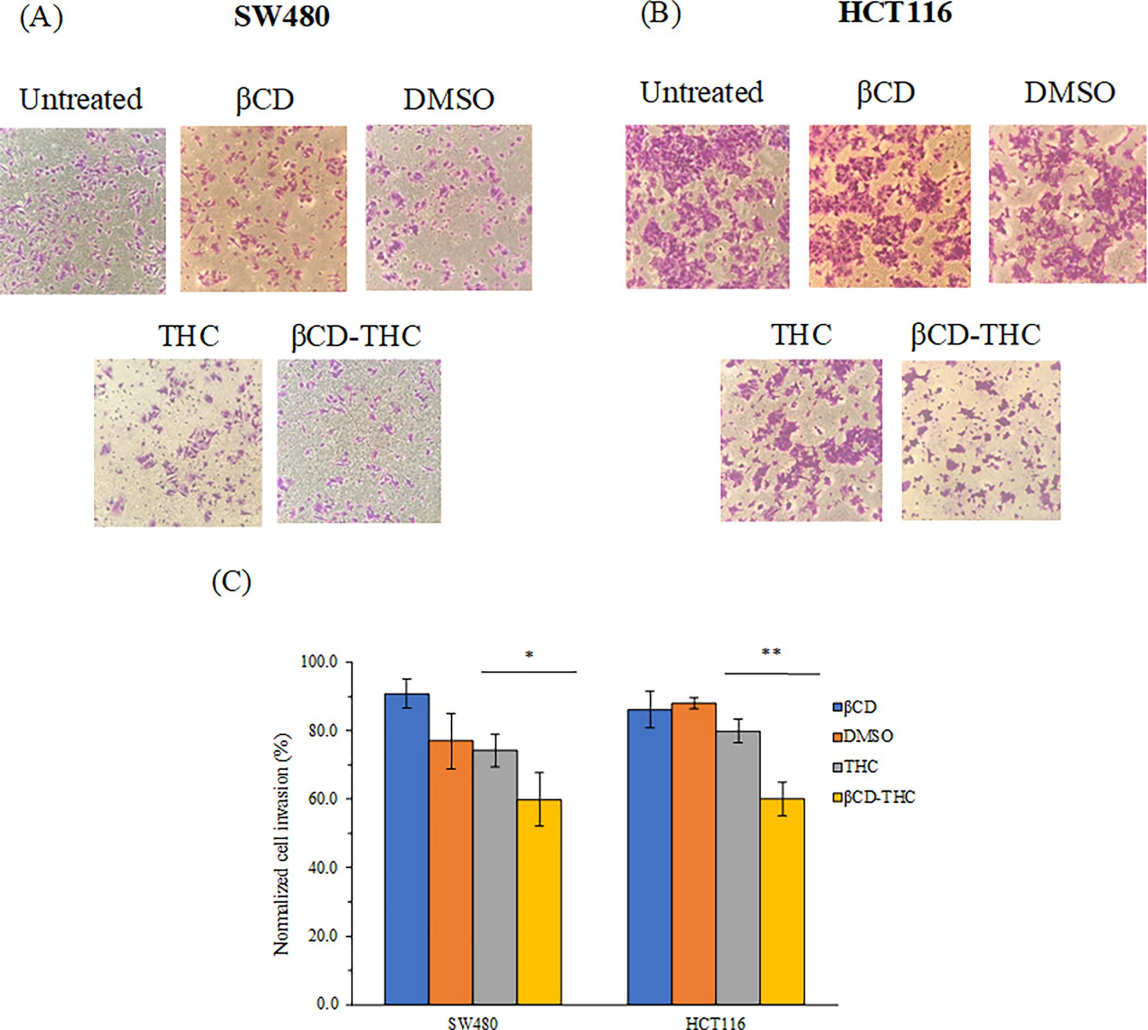
Comparison of invasion properties of (A) SW480 and (B) HCT116 cells upon treatment with THC, βCD, βCD-THC inclusion complex and DMSO solvent controls for 24 h. Images were captured at 200x total magnification after being stained with crystal violet on the bottom membrane of the Matrigel transwell invasion insert. (C) The relative percentages of invaded cells per field are expressed in mean ± standard deviation from three independent replicates, denoted with a *p < 0.05 and **p < 0.01 for the significant differences between THC and βCD-THC inclusion complex.

Cell penetration was mediated by the invasion of underlying cells transmigrating through the Matrigel. The stained cells by crystal violet under 200x magnification were observed under an inverted microscope (Fig 8A). After 24 h of incubation time, the βCD-THC inclusion complex treated group demonstrated a significant decrease (*p* < 0.05 for SW480 cells and *p* < 0.01 for HCT116 cells) in invasion properties in comparison with the THC treated group (Fig 8B). According to the results obtained for both cell lines, the cell invasion capabilities decreased when compared between THC and βCD-THC inclusion complex, from 75.5 to 67.8% (SW480 cells) and 79.8 to 59.7% (HCT116 cells). Additionally, DMSO acting as the solvent control in dissolving THC together with βCD showed a neglectable effect on cell invasion levels. This thereby confirms the inclusion complex as a superior formulation compared to standalone THC. The present data reinforces the application of the drug in CRC against metastatic progression through inhibitory effects on cell adhesion and invasion properties [42].

### Apoptosis-mediated cell death evaluation by double Annexin V/PI staining

**Fig 9:**
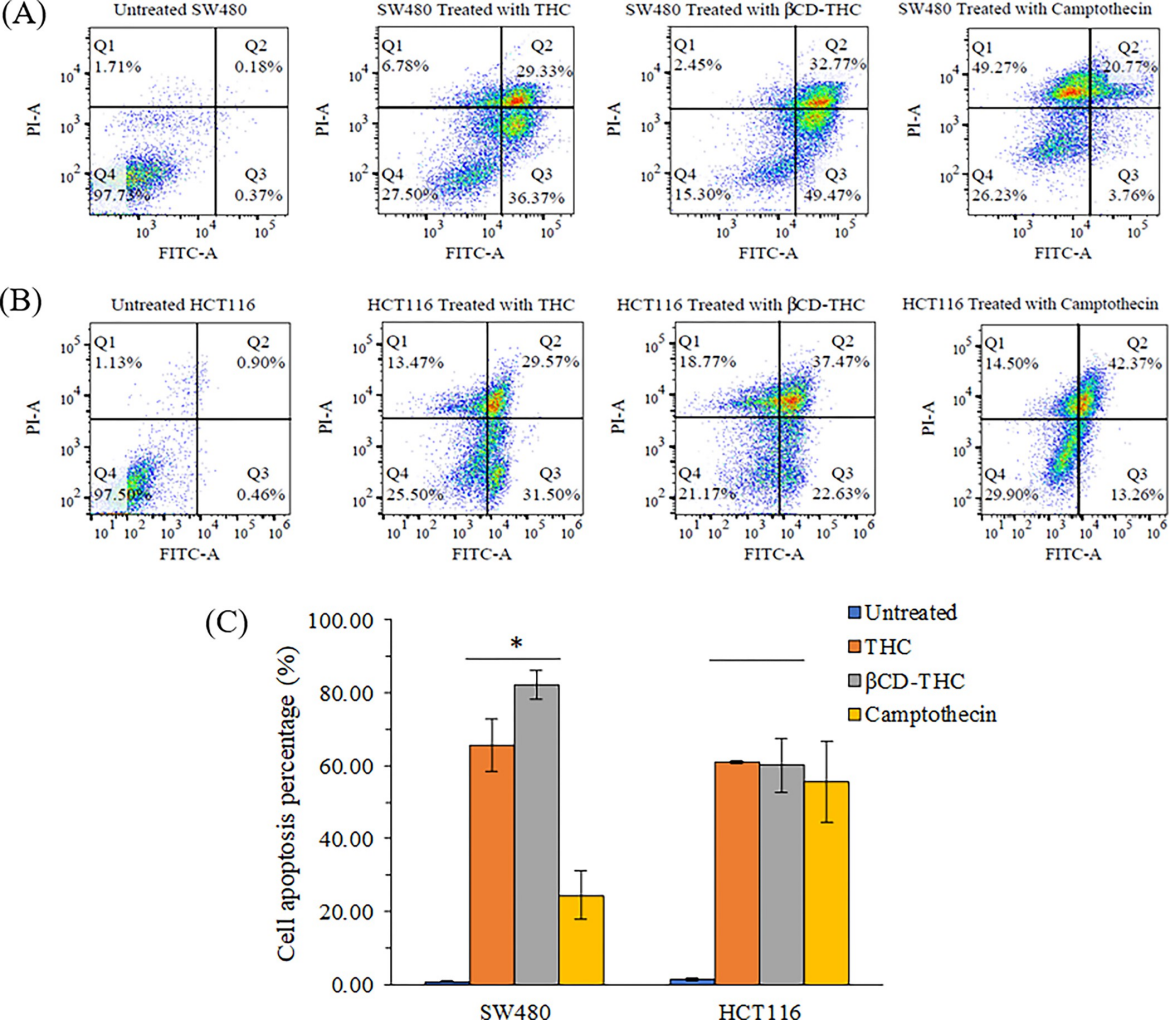
Flow cytometry analysis of Annexin V/FITC-PI on SW480 and HCT116 cells treated with negative control, THC, βCD-THC inclusion complex, and camptothecin. (A) Dot plot of SW480 cells with different treatments. (B) Dot plot of HCT116 cells with different treatments. Each representative dot plot is divided into Q1, necrosis; Q2, late apoptosis; Q3, early apoptosis and Q4, viable cell. (C) Quantitative percentages of apoptotic cell populations after treatment. Error bars represent the standard deviation of the mean from three replicates. Significant differences were denoted as *p < 0.05 against THC and βCD-THC inclusion complex treatment groups.

Apoptosis was indicated by Annexin V/PI double staining to detect the externalization of phosphatidylserine on the cell membrane and the PI-specific stained cell nuclei after rupturing of the membrane [43]. For SW480, THC demonstrated an increase in early and late apoptotic cells of 36.37% and 29.33%, respectively (Fig 9A). Similar patterns of apoptotic induction of THC were also observed on SW480 and HCT116 at 65.70% and 61.07%, respectively. In addition, the βCD-THC inclusion complex significantly increased (*p* < 0.05) the cell apoptotic rate by about 82.23% in comparison to THC on SW480 cells (Fig 9B). However, the observed effect of the βCD-THC inclusion complex on HCT116 cells was slightly skewed towards late apoptosis at 37.47% compared with THC. In the control group, cell apoptosis was only 0.55% and 1.36% for SW480 and HCT116 cells, respectively, whereas camptothecin-treated cells showed 24.53% and 55.63%, respectively.

### Western blot analysis of PARP cleavage following THC treatment

**Fig 10:**
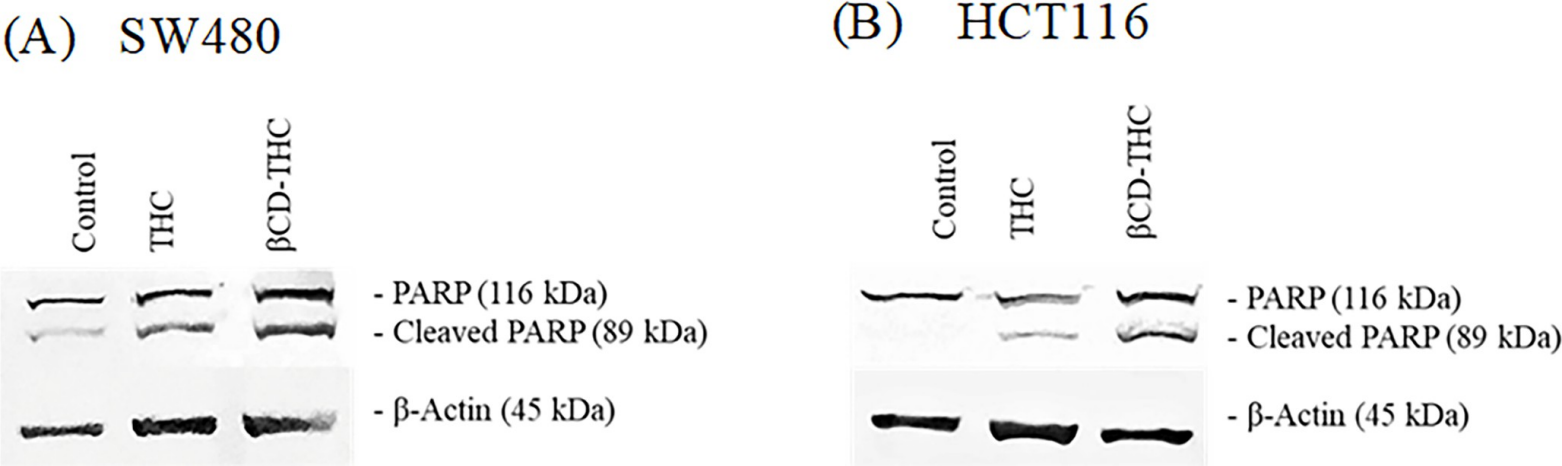
Confirmation of apoptosis-mediated cell death through the activation of caspase-3 in (A) SW480 and (B) HCT116 cells. The western blot analysis showed the detection of cleaved PARP (89 kDa) large fragment and full-length PARP fragment (116 kDa), while β-actin functioned as a loading control.

**Fig 11:**
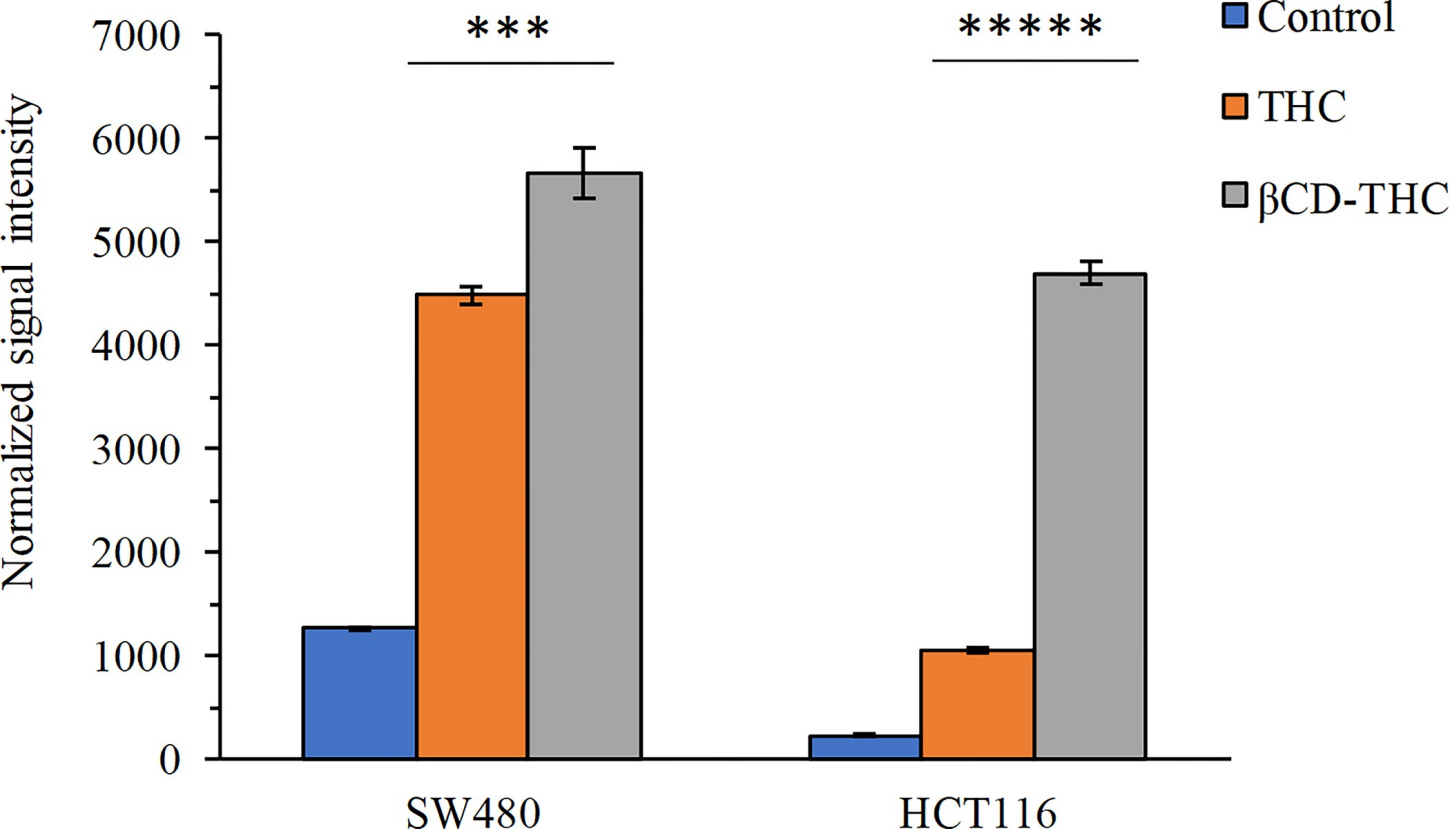
Cleaved PARP normalised to β-actin. Data for cleaved PARP were presented as mean ± S.D. of three independent replicates. Statistically significant changes between THC and βCD-THC groups are denoted ****p < 0.0001 and *****p < 0.00001.

The mitochondria-mediated intrinsic pathway and the death receptor-mediated extrinsic pathway are the two central pathways that play a vital role in inducing cell apoptosis among caspase family members. To further confirm that caspase 3, the central executor involved in apoptosis [44] was activated, a western blot analysis was carried out to indicate the proteolytic cleavage of PARP by caspase 3. The cleavage of PARP (116kDa to 89 kDa) was shown in Fig 10 after treatment with THC and βCD-THC inclusion complex, while the control vehicle treatment showed significantly less or no cleavage of PARP bands (Fig 11). These observations were presented as a strong hallmark of apoptotic events.

### *In vivo* anti-tumour effects

**Fig 12:**
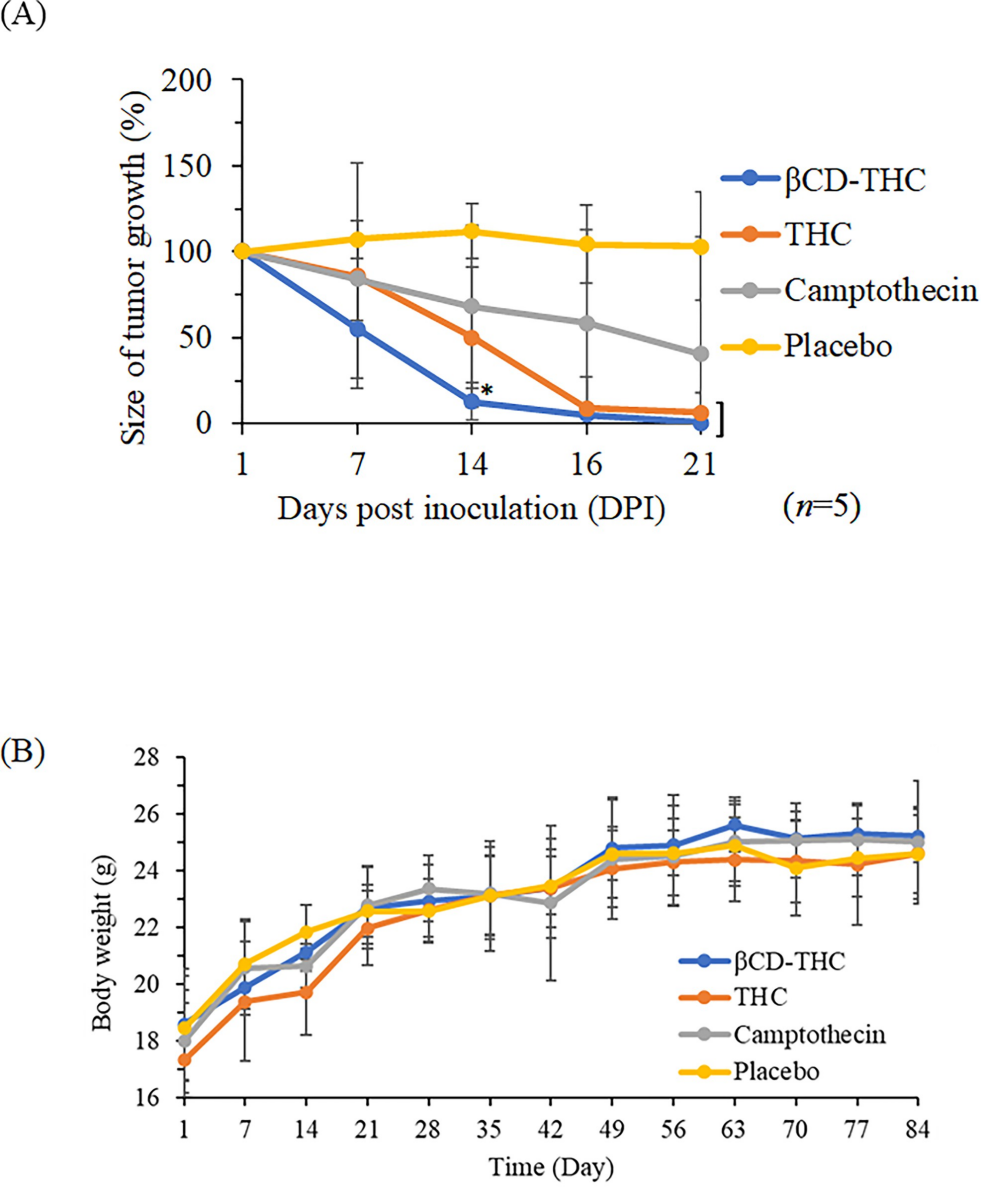
The impact of various treatment regimens on SW480 tumour reduction in BALB/C nude mice is evident. (A) THC group and representative photographs (*n* = 5) showcase tumours harvested 26 days post-implantation. (B) Evaluation of body weight loss among treatment groups on SW480 xenografts. The placebo refers to groups treated with a 0.9% (w/v) NaCl solution, while the treatment initiation occurred 3 weeks post-tumour implantation.

The physiological outcome of the inclusion complex, comparable with its standalone, was investigated *in vivo* on the growth of implanted SW480 colorectal tumour xenograft models (Fig 12). NaCl was used as a placebo instead of DMSO at injection volumes of 100μl because athymic BALB/C nude mice were sensitive to DMSO which could cause hindlimb paralysis [45]. The estimated safe dose of biweekly drug administration was calculated based on IC_50_ from *in vitro* experiments and normalised against mice body weight. No adverse effects were noted on the drug administration up to 10g for THC, with only minimal headaches or mild diarrhoea observed [46]. Only placebo-treated mice exhibited body weight loss, while all other therapeutic groups maintained a similar increase in body weight (Fig 12B).

Colorectal tumour development was monitored on days 7, 14, 16, and 21 after tumour cell xenograft (day 14: *p <0.05) (Fig 12A). Across all tested βCD-THC inclusion complex treatments, significant tumour volume regression was achieved compared to the placebo, standalone THC, and the CPT positive control groups. Interestingly, on day 14, tumour incidence rates were below 15% in the group treated with βCD-THC, intermediate (50–60%) in mice treated with THC standalone and 100% in placebo groups. During necropsy procedures, no other undesired histopathological signs on significant organs, such as the liver and kidneys were observed. As a result of THC’s capacity to target tumours, it was determined that the inflammatory and physiological effects of the inclusion complex were less than those of CPT medicines.

### Reduction of CEA tumour marker levels *in vivo*

**Fig 13:**
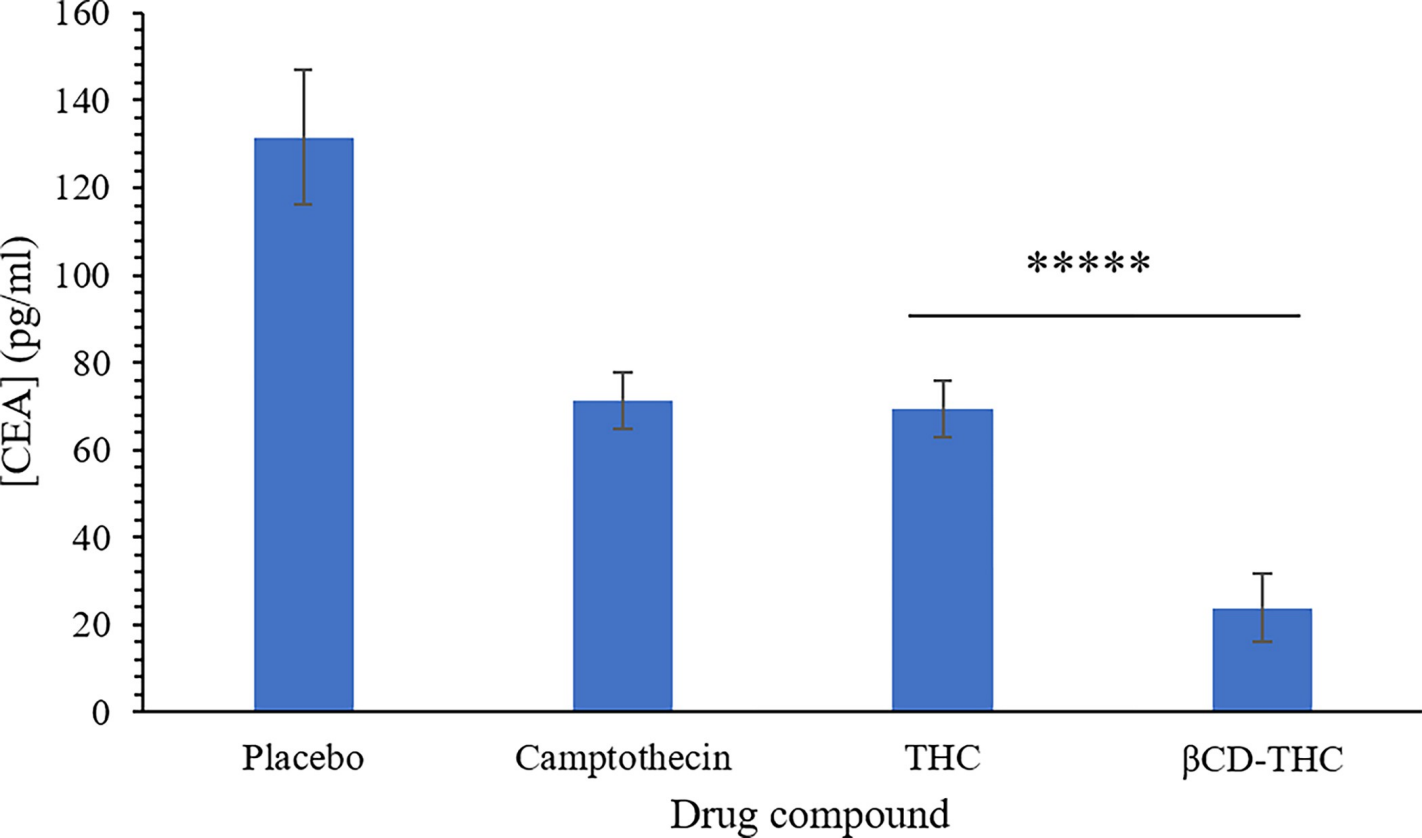
CEA tumour antigen marker levels of SW480 human colorectal tumour xenografts. βCD-THC inclusion complex in comparison to placebo, CPT, and THC alone. All values are shown as mean ± standard deviation. Statistically significant changes in comparison between inclusion complex and drug standalone are denoted *****p < 0.00001.

The effectiveness of the treatment was assessed using sandwich ELISA targeting tumour antigen markers. According to Fig 13, CEA levels consistently decreased by 81.9% upon treatment with βCD-THC inclusion complex compared with the untreated group, while THC standalone showed a 47.2% reduction against the placebo group. Researchers have reported that patients who received THC in chemotherapy and radiotherapy treatment also resulted in improvement of CRC treatment effectiveness [47]. Notably, the reductions in tumour marker levels were transient, rebounding swiftly upon the cessation of treatment, in line with changes in tumour bulk volume observed across all treated groups.

### Bioactivity of THC on CYP1A2

**Fig 14:**
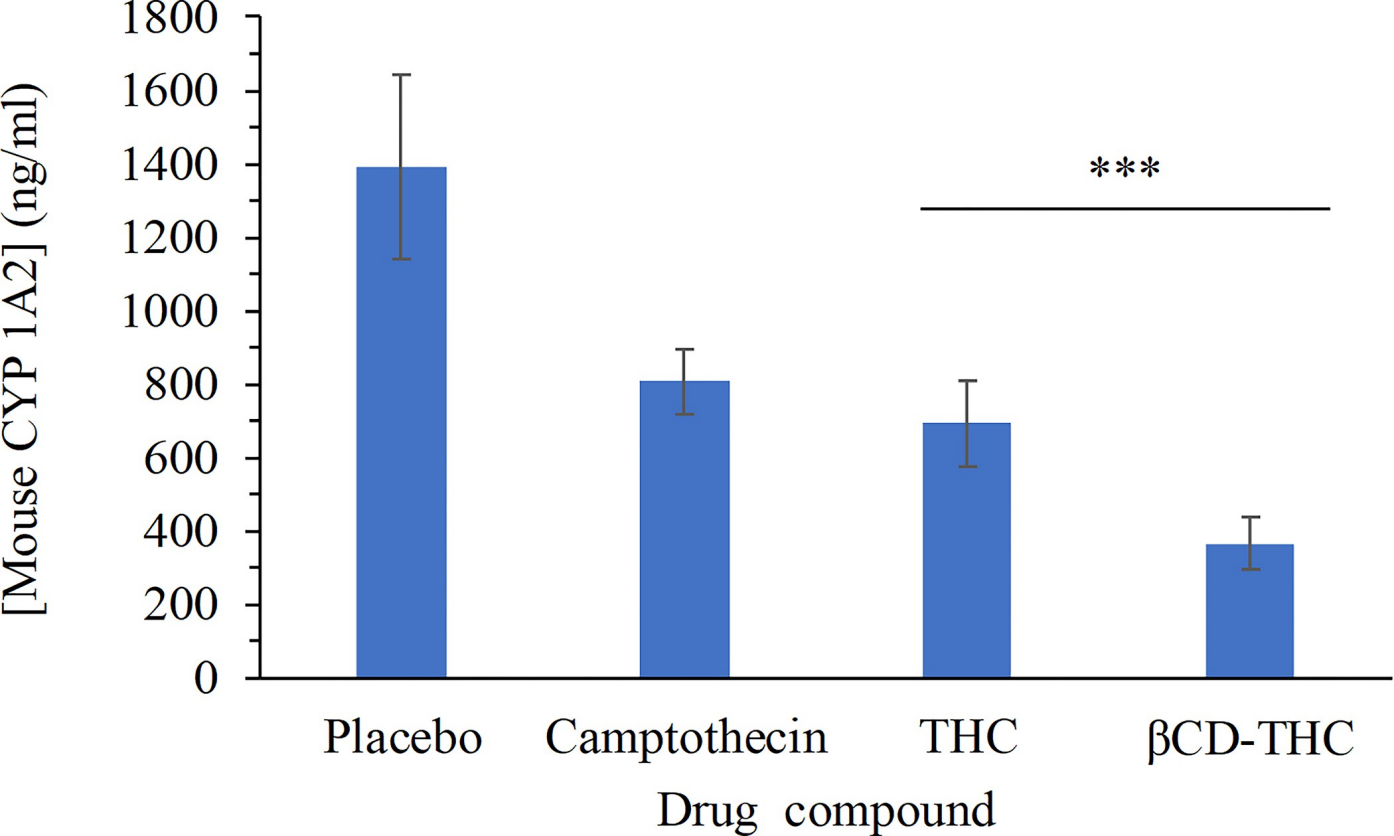
Effect of curcuminoids on CYP21A2 activity. Human CRC cells, SW480 were used as a source of mouse CYP21A2 activity. The liver section on Nu/Nu nude mice was used to conduct the analysis Data shown here are from the mean and standard deviation (n = 5) giving significant results when *** *p* < 0.001.

The effects of THC on CYP1A2 remain unclear, and further exploration is needed. In Fig 14, a correlation between both elevated and reduced CYP1A2 enzyme activity and an augmented risk of cancer was observed. In individuals overexpressing CYP1A2, long-term THC administration may inhibit the activation of toxic compounds by CYP1A2, potentially reducing the risk of CRC [48, 49]. However, some of the curcuminoid degradation products can have toxic side effects [50]. As shown in Fig 14, THC showed a significant difference (βCD-THC, *p* < 0.001) in comparison with the inclusion complex. The readings showed that mouse CYP1A2 levels reached almost 1,400 ng/ml without any treatment, compared with those treated with THC, 693.02 ng/ml and βCD-THC, 365.43 ng/ml. These findings thereby proved the importance of compound encapsulation that can consequently decrease CYP1A2 enzyme activity. While the benefits of THC on the human body are clear, the *in vivo* stability of curcuminoids remains a concern. To overcome this issue, enhancing bioavailability and stability through sustained-release nanoparticles or liposome preparations shows promise for their application in various disease models [11].

### Histopathology of liver and kidney section

**Fig 15:**
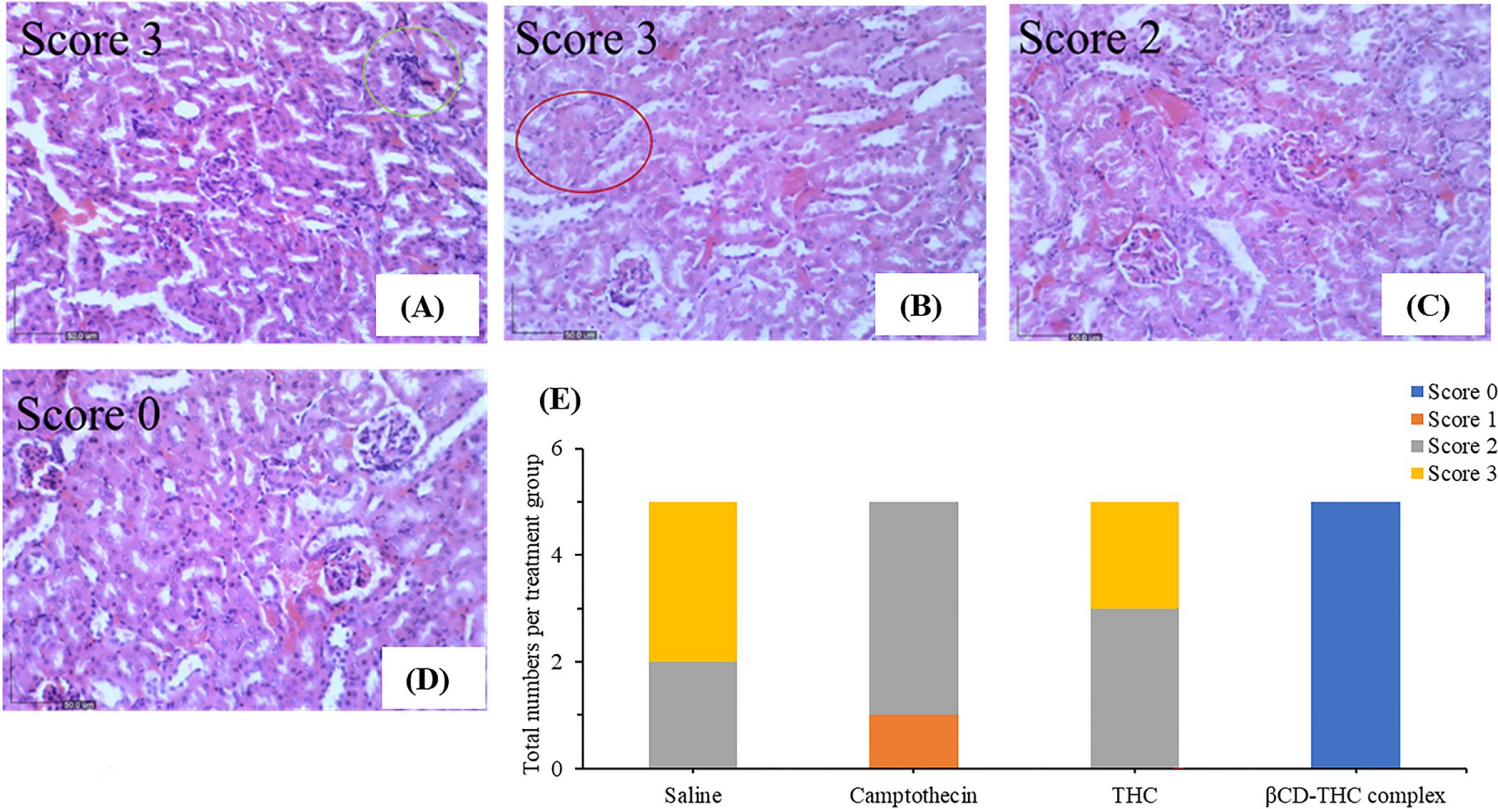
H&E staining of kidney tissue extracted from nude mice xenografts at 200× magnification. A) abnormal histological appearance of the kidney in the control group, such as poorly differentiated morphology of glomerulus surrounded by inflammatory cell infiltrates (green elliptic curve). Thrombosis was also examined in the tissue section as presented by vascular injury. B) CPT injury as observed through glomerular basement membrane thickening and red stained cytoplasm (red elliptic curve) C) Feature of glomerular hypercellularity, enlarged tubular epithelial cells and mild mononuclear inflammatory aggregation were observed in the THC treatment group. D) Kidney architecture was well-distributed with no fibrosis on the βCD-THC complex group. E) Stack bar graph summarizing numbers of neonates with 0–3 scores based on treatment groups. Score 0- normal, 1- mild, 2- moderate, and 3- severe.

**Fig 16:**
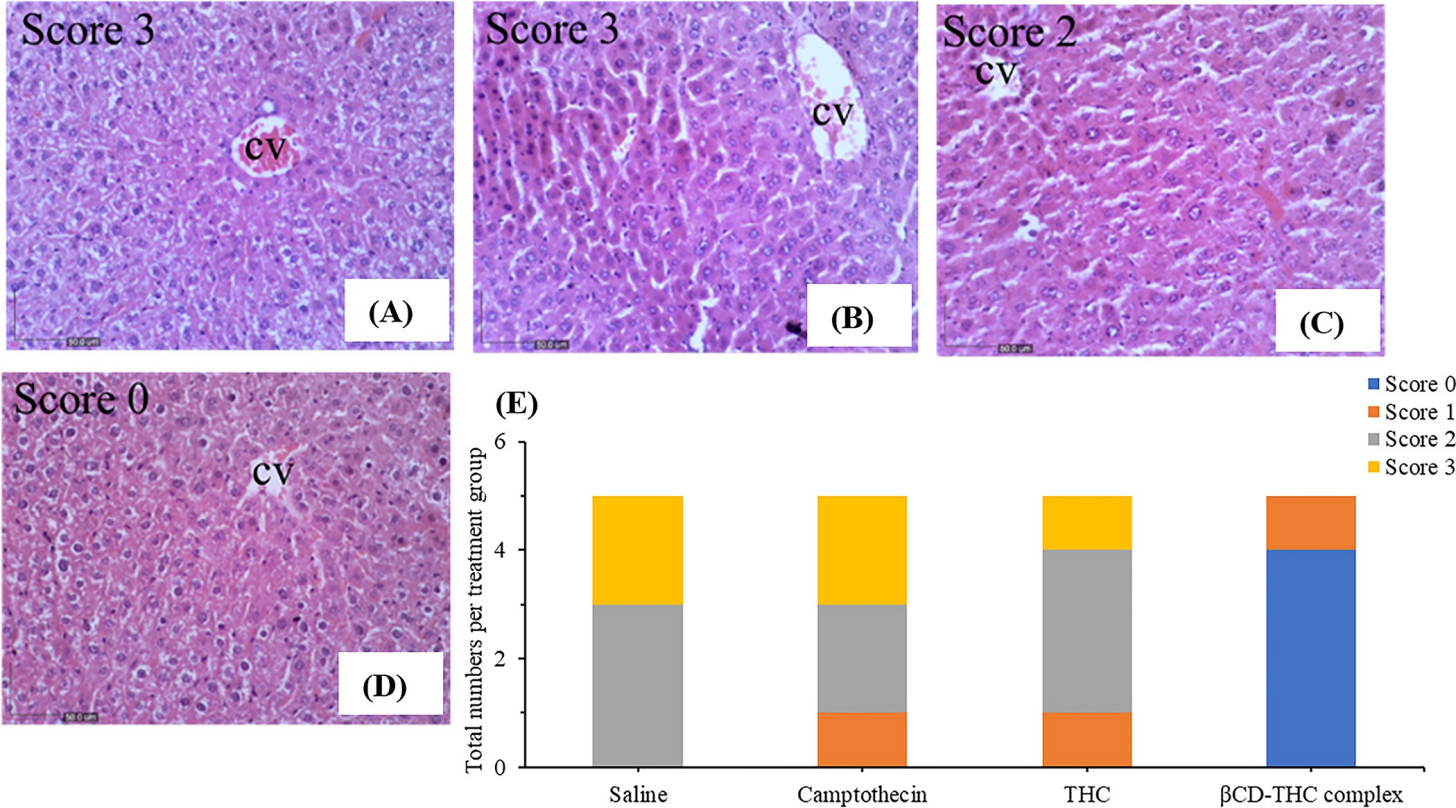
Representative photomicrographs of liver histopathology (200×). (A) liver section from control BALB/C nude mice showing metastasis injury with zone 3 necrosis around the central vein (CV). Numerous red blood cells at the inner part of the CV are seen with little lobular inflammation. (B) The liver of mice after *in situ* subcutaneous injection of CPT (2 mg/kg) showed congested blood sinusoids with nuclear abnormalities, such as hyperchromasia. There was also a collection of inflammatory cells with several hepatocytes that fit the morphological criteria of apoptosis. (C) THC-treated (44mg/kg) mice liver section showed cellular lesions and minimal degenerations. D) High dose treatment of 312mg/kg of βCD-THC complex on mice liver showed significant protection against drug-induced hepatic injury. E) Stack bar graph summarizing numbers of neonates with 0–3 scores based on treatment groups. Score 0- normal, 1- mild, 2- moderate, and 3- severe.

Histological analysis involving H&E staining to examine the cytotoxic effects of standalone THC and βCD-THC on kidney and liver tissues is described in this section. The accumulation of drugs and metastasis of malignancies to the liver is especially prominent for gastrointestinal cancers such as CRC owing to the direct flow of blood through the portal vein [51–53]. CRC often leads to liver metastases in approximately 60% of affected patients [54]. Therefore, histological and pathological alterations in these organs often serve as indicators of drug-induced injury [55] and potential bioaccumulation [56, 57]. Kidney and liver sections from xenograft mice treated with βCD-THC exhibited a normal architecture of hepatic and renal cells with well-preserved cytoplasm and no significant morphological changes compared to the placebo and CPT control group (Figs 15 and 16).

## Conclusion

The encapsulation of THC in inclusion complexes resulted in significantly improved aqueous dispersion, drug content, and release profiles. This led to enhanced delivery of drugs and improved anticancer effects compared to pure non-complexed drugs. Subsequent evaluations not only affirmed its enhanced solubility but also highlighted its potential use to inhibit angiogenesis-based regulation, tumour growth and metastasis compared to standalone drugs. This study validates the possibility of increasing THC solubility using an inclusion complex for CRC therapy with fewer undesirable side effects. Overall, the outcome of this study provides widespread attention for future investigation and development aimed at maximizing the therapeutic benefits of curcuminoids as well as the scope of CD-based drug delivery systems with desired properties in the pharmaceutical industry.

## Supporting information

S1 Raw image (PNG)

S2 Raw image (PNG)

S3 Raw image (TIFF)

S4 Raw image (TIFF)

S5 Raw image (TIFF)

S6 Raw image (TIFF)
