# Supplementary figures and images for: Enhancing the solubility and potency of tetrahydrocurcumin as an anti-cancer agent using a β-cyclodextrin inclusion complex approach
Source: PLoS One. 2024 Jul 26;19(7):e0305171. doi: 10.1371/journal.pone.0305171 (PMC11280155; doi:10.1371/journal.pone.0305171)

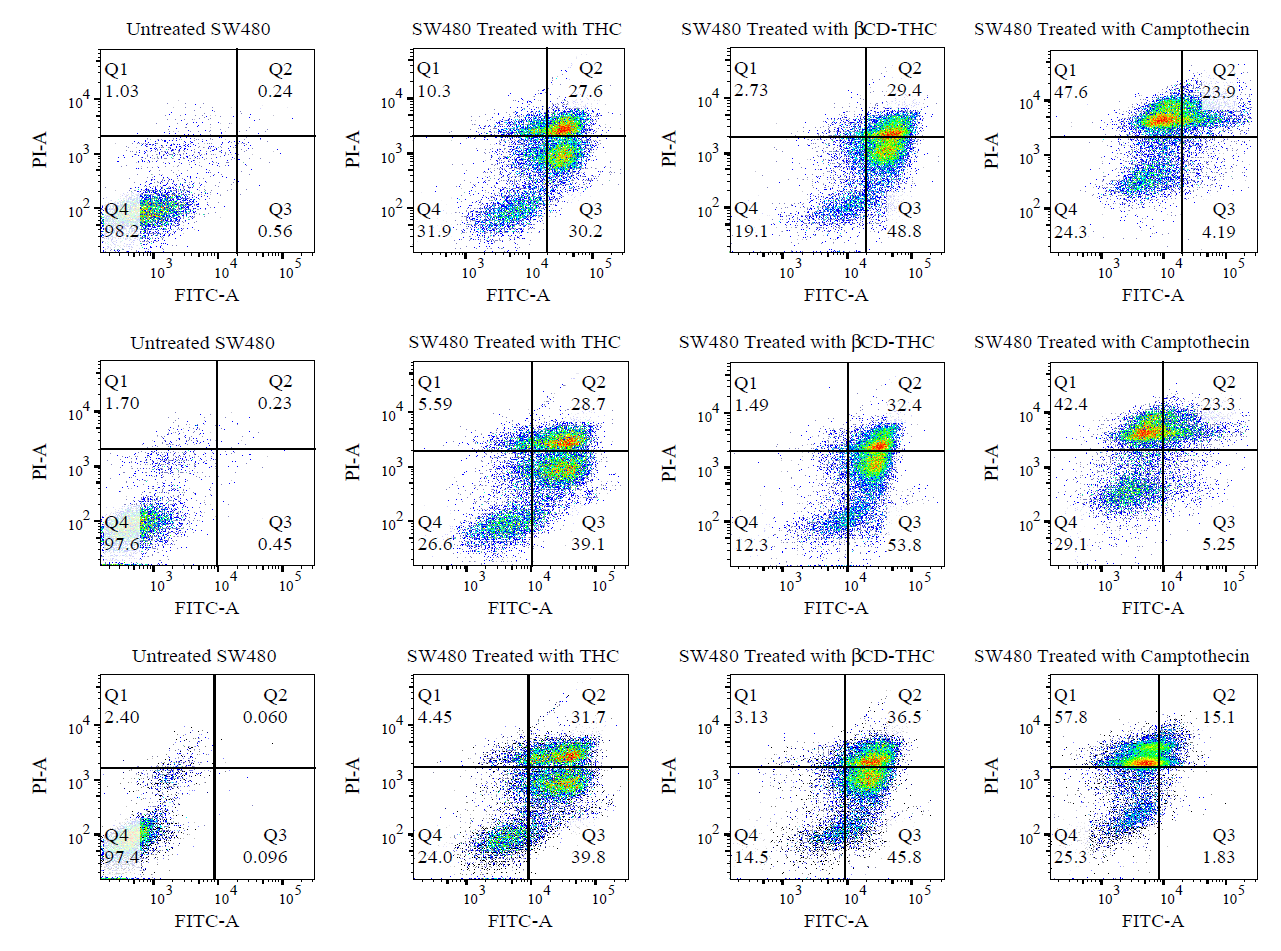

Supplement: S1 Raw image — (PNG) [file pone.0305171.s001.png]

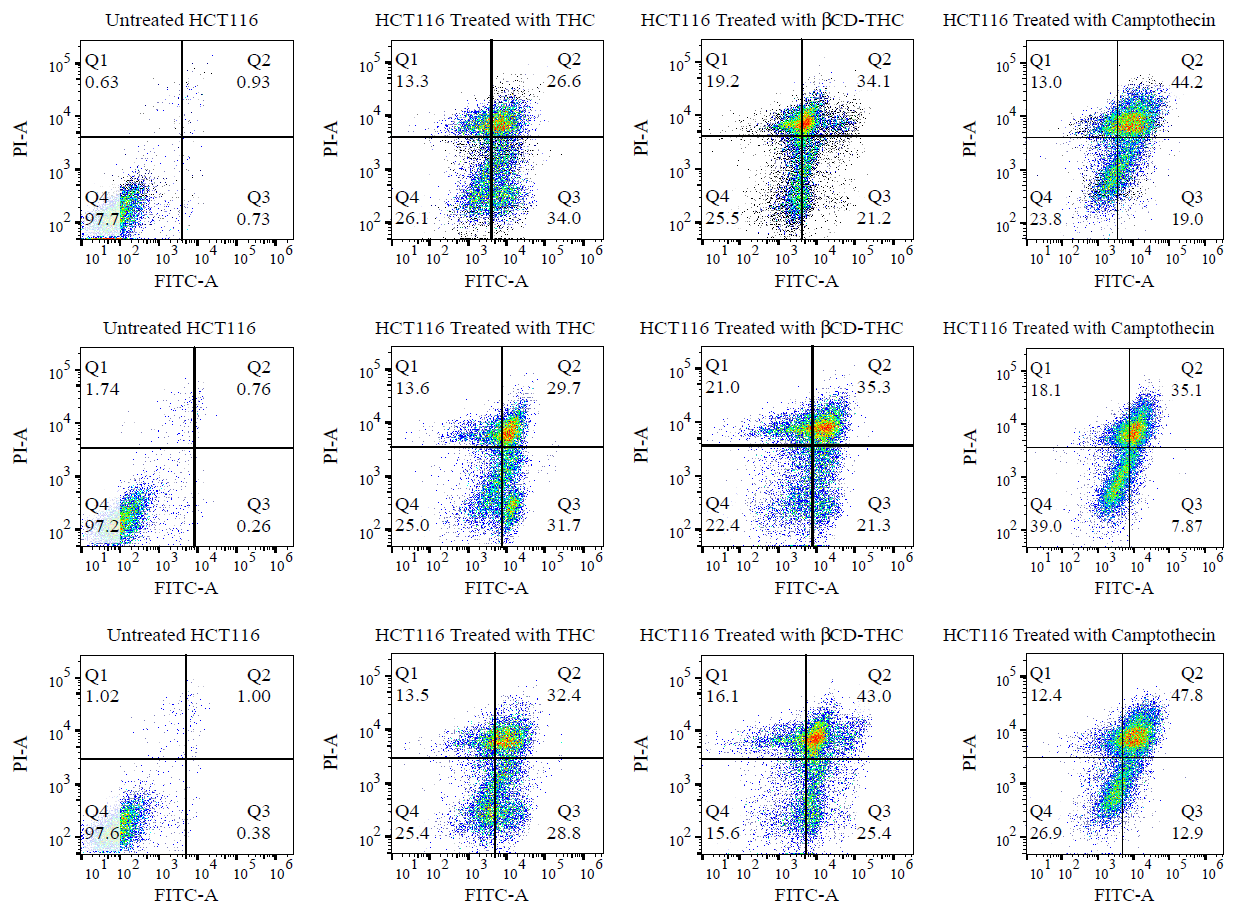

Supplement: S2 Raw image — (PNG) [file pone.0305171.s002.png]

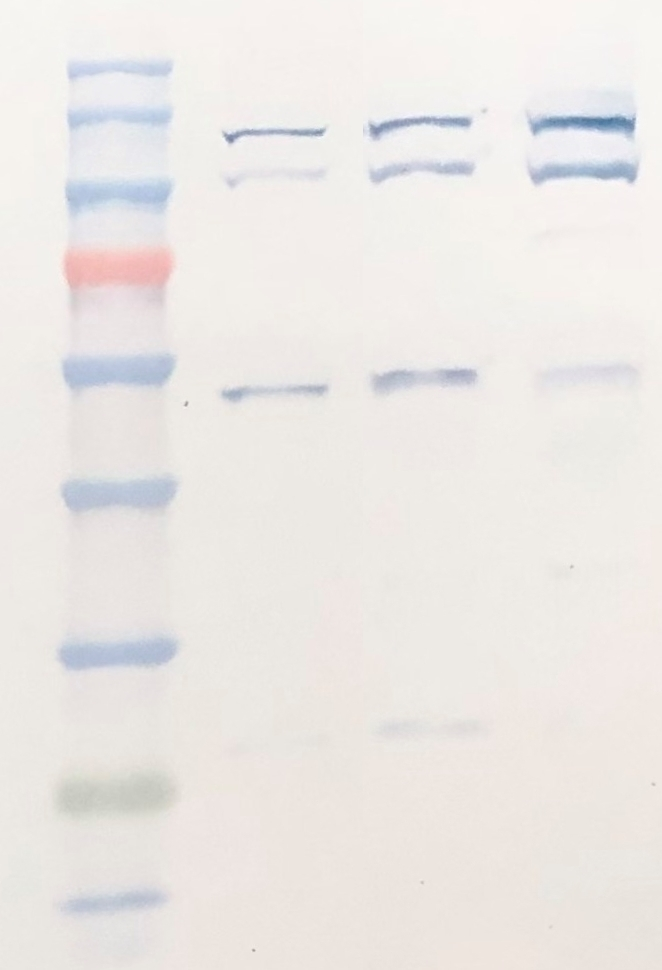

Supplement: S3 Raw image — (TIFF) [file pone.0305171.s003.tiff]

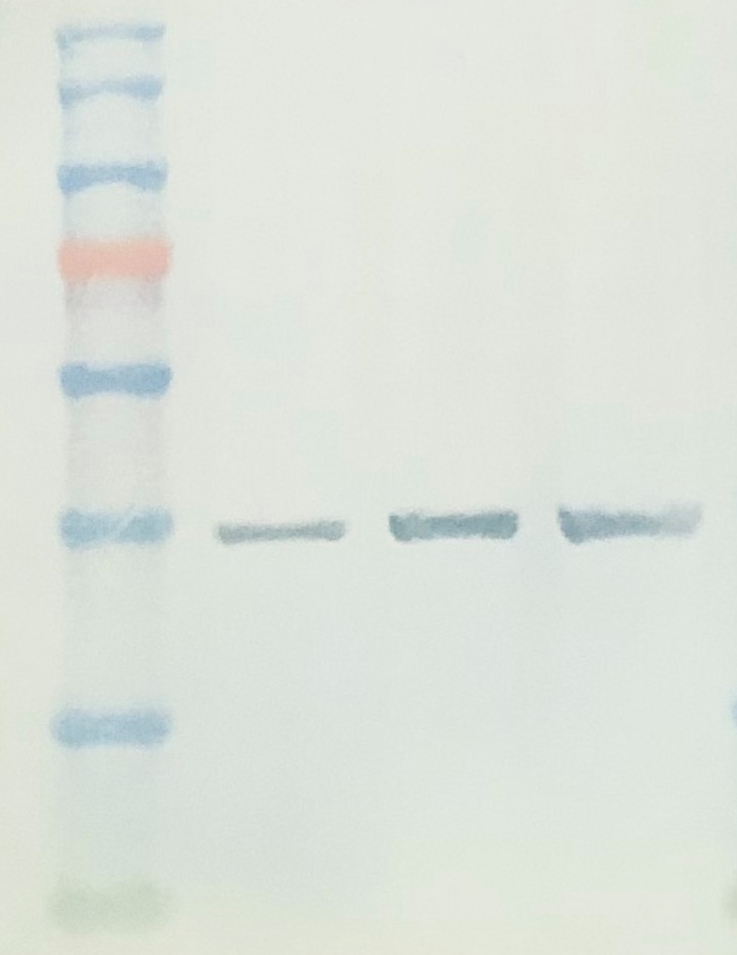

Supplement: S4 Raw image — (TIFF) [file pone.0305171.s004.tiff]

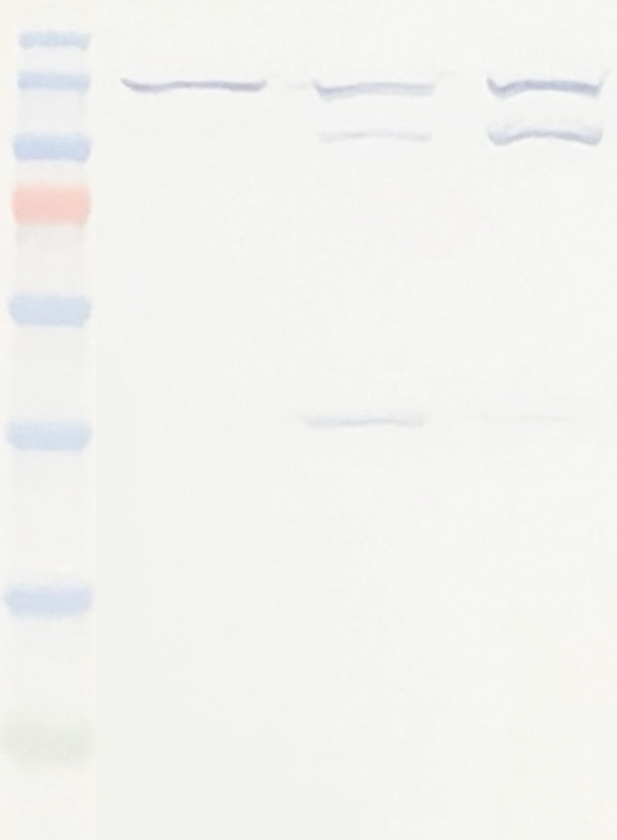

Supplement: S5 Raw image — (TIFF) [file pone.0305171.s005.tiff]

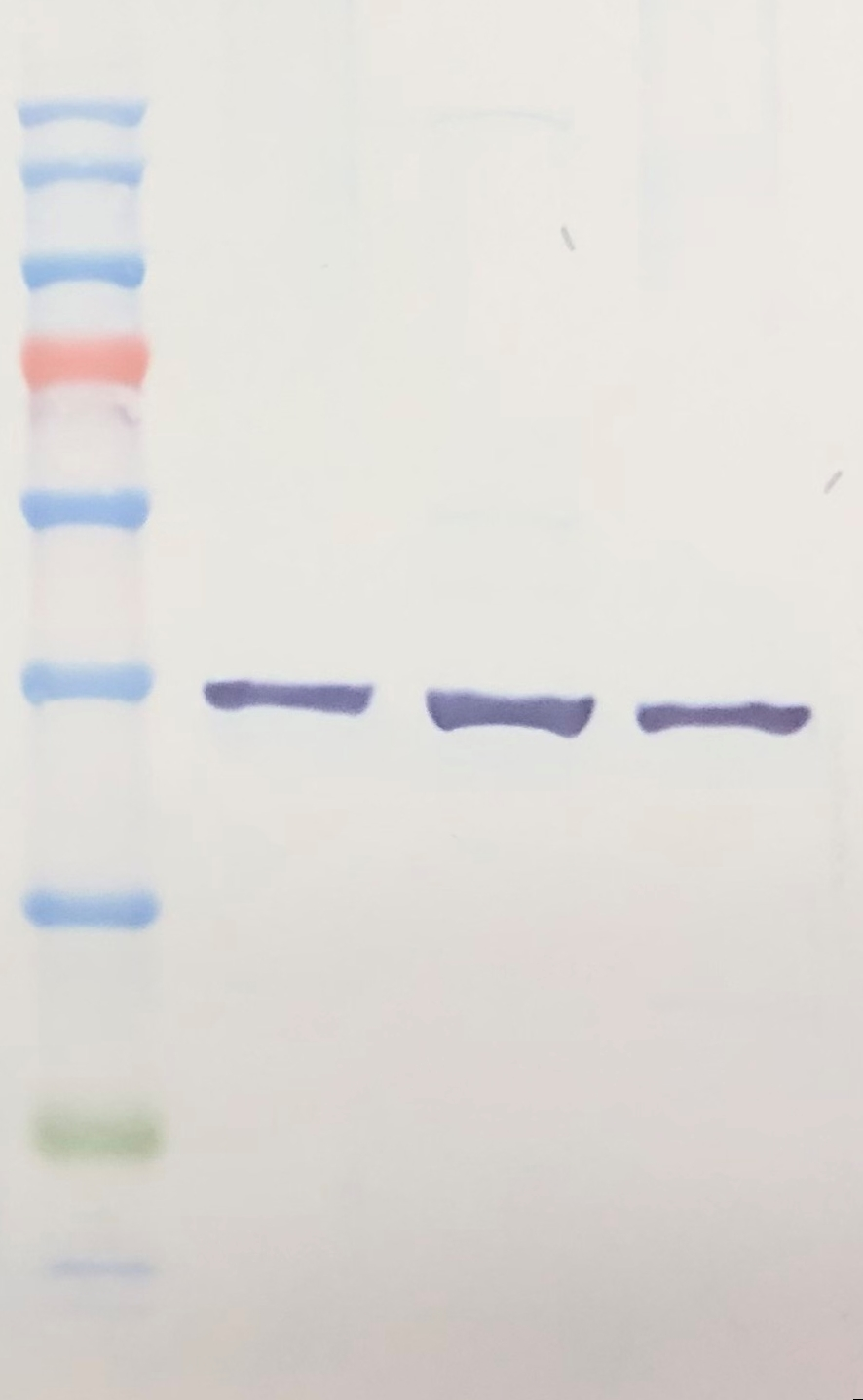

Supplement: S6 Raw image — (TIFF) [file pone.0305171.s006.tiff]
